# Resolution-adapted recombination of structural features significantly improves sampling in restraint-guided structure calculation

**DOI:** 10.1002/prot.23245

**Published:** 2011-11-09

**Authors:** Oliver F Lange, David Baker

**Affiliations:** 1 Department Chemie, Biomolecular NMR and Munich Center for Integrated Protein Science, Technische Universität MünchenGarching, Germany; 2 Institute of Structural Biology, Helmholtz Zentrum MünchenNeuherberg, Germany; 3 Biochemistry Department, University of WashingtonSeattle; 4 Howard Hughes Medical Institute, University of WashingtonSeattle

**Keywords:** sparse restraints, sparse NMR data, structure prediction, structure refinement, *de-novo* structure calculation, enhanced sampling methods, genetic algorithms, optimization

## Abstract

Recent work has shown that NMR structures can be determined by integrating sparse NMR data with structure prediction methods such as Rosetta. The experimental data serve to guide the search for the lowest energy state towards the deep minimum at the native state which is frequently missed in Rosetta *de novo* structure calculations. However, as the protein size increases, sampling again becomes limiting; for example, the standard Rosetta protocol involving Monte Carlo fragment insertion starting from an extended chain fails to converge for proteins over 150 amino acids even with guidance from chemical shifts (CS-Rosetta) and other NMR data. The primary limitation of this protocol—that every folding trajectory is completely independent of every other—was recently overcome with the development of a new approach involving resolution-adapted structural recombination (RASREC). Here we describe the RASREC approach in detail and compare it to standard CS-Rosetta. We show that the improved sampling of RASREC is essential in obtaining accurate structures over a benchmark set of 11 proteins in the 15-25 kDa size range using chemical shifts, backbone RDCs and H^N^-H^N^ NOE data; in a number of cases the improved sampling methodology makes a larger contribution than incorporation of additional experimental data. Experimental data are invaluable for guiding sampling to the vicinity of the global energy minimum, but for larger proteins, the standard Rosetta fold-from-extended-chain protocol does not converge on the native minimum even with experimental data and the more powerful RASREC approach is necessary to converge to accurate solutions.

## INTRODUCTION

NMR structure determination is challenging for proteins above 15 kDa. Spectral overlap and slow rotational tumbling lead to ambiguity in the NOESY-derived distance restraints. These problems can be partially overcome by deuteration, but then fewer proton spins are available to report on distances. Obtaining accurate structures from the resulting sparse datasets is an important current challenge.

Supplementing sparse restraints with the detailed physical-chemistry captured by structure prediction methods such as ROSETTA holds promise to achieve accurate structures for higher molecular weight proteins.^1^–^4^ However, *de-novo* structure prediction sampling methodologies are also challenged as protein size and complexity increases.^5^, ^6^ The first structures solved with chemical-shift based methods were all smaller than 15 kDa with relatively low contact order,^1^, ^7^ (the average separation in sequence of contacting residues).^8^ Generally ROSETTA *de-novo* structure calculations run into sampling issues for proteins with more than 100 amino acids, and the success rate is also reduced for high-contact order structures.^1^, ^9^ We have shown that additional sparse NMR data—chemical shifts, RDCs, and backbone H^N^-H^N^ contacts—can guide sampling towards the native structure, and thus help ROSETTA to overcome some of the sampling issues.^1^, ^10^ However, this experimental guidance only increases the size limit slightly—to 120–130 amino acids—and hence the original CS-Rosetta protocol does not have a robust success rate for proteins over 15 kDa.^10^

To overcome these size limitations we have developed an iterative sampling protocol that recombines structural features found in intermediate structures. The new resolution-adapted structural recombination (RASREC) protocol combines the strengths of previous approaches to optimization in Rosetta. Bradley *et al*. sought to overcome the difficulty in *de novo* structure prediction of nonlocal beta sheet topologies by resampling beta pairings using broken chain folding kinematics which held the desired pairings in place.^11^ Blum *et al*. similarly used energy and occurrence frequency to identify, from an initial round of Rosetta models, strand pairings, and local structures likely to be present in the native structure and then generated models enriched in these features in a second round.^12^ Brunette *et al*. identified models present early in Rosetta trajectories which gave rise to low energy all atom structures, and restarted additional trajectories from these promising starting points.^13^, ^14^ Qian *et al*. developed an iterative approach to refining starting models with the correct topology by alternately rebuilding and refining regions of the structure that differed the most in the ensemble.^15^

As reported previously, using a beta-version of RASREC, we were able to push the size limit for CS-Rosetta structures well above the >15 kDa range using backbone NOE and RDC data.^10^ Here we provide a detailed characterization of the new method and demonstrate that there is strong synergy between recombinant iterative sampling and sparse NMR data. The method should also be effective with other types of sparse structural data.

## METHODS

### Resolution-adapted structural recombination

For larger proteins (>15 kDa), in particular those with a high-contact order, the *de-novo* fragment assembly protocol of ROSETTA generally fails to converge on the native fold. Nevertheless, even in unsuccessful structure calculations native structural features tend to occur frequently. Experimental restraints further facilitate enrichment of these features. The RASREC protocol seeks to improve sampling close to the native structure by recombination of frequently occurring structural features. The structural features we focused on are beta-strand topologies, short contiguous stretches of backbone conformation (fragments), and noncontiguous collections of secondary structure elements, that is, a protein core.

We found it important to adapt feature-resampling to the resolution of the intermediate ensemble. At the beginning of the calculations when the precision (i.e., resolution) is still very low, a coarse-grained featurization of protein structure is required. We found a description that focuses on beta-strand topologies, that is, strand-pairings and their register, to be helpful. In the intermediate resolution range, we resample from early stage models which have given rise to low energy final models,^14^ which we call *proto-fold* resampling. Such resampling allows changes on the fold level, but biases the search to (kinetically) related folds. In the last stage we refine the protein to high-resolution and restrict aggressive sampling to the loops while the core regions can plastically adapt to accommodate changed loop-conformations. This stage, similar to the previous rebuild-and-refine protocol^15^ requires a well-defined and near-native core to work, and thus hinges on the convergence of the mid-stage protocol.

The RASREC protocol has six Stages (I–VI), an initial exploration stage and five resampling stages. The first four stages use the ROSETTA low-resolution energy, the remaining two stages use ROSETTA all-atom energy. For both modes, low-resolution and all-atom, we maintain a pool of the 500 best scoring decoys. Additional energy terms based on prior knowledge or experimental data can be used. The acceptance rate into the pool is recorded and when below 10%, the respective resampling stage is terminated. The structure pool determines the features for the next generation of decoys during resampling Stages II–VI.

To maintain sufficient diversity in the structural pool we require in Stages IV, V, and VI that newly generated structures are at least 2.0, 1.5, and 1.5 Å Ca-RMSD away from structures that existed in the pool at the start of a given trajectory. A freshly generated structure that is closer than this threshold to a structure in the pool will be swapped for this structure if it has a lower energy.

**Figure 1:**
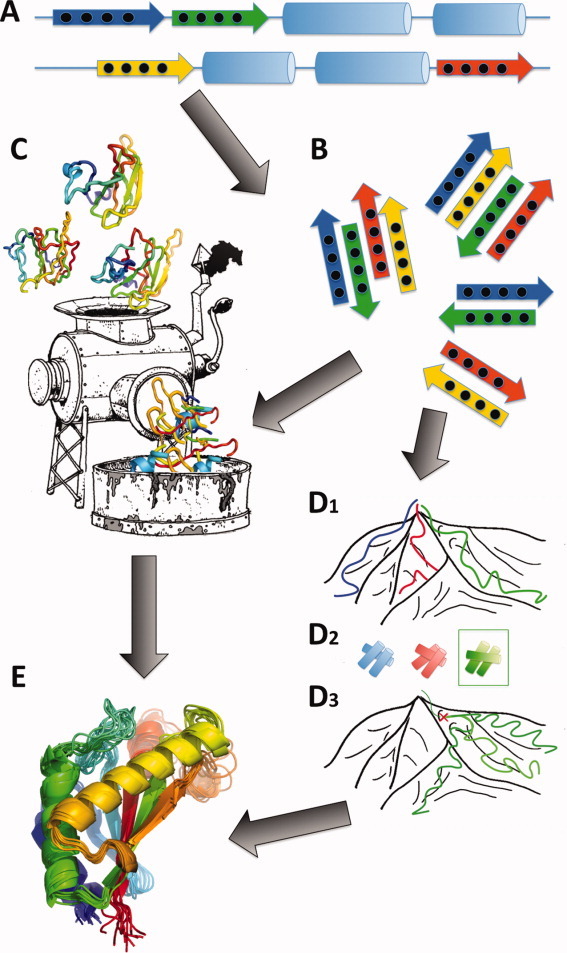
Illustration of RASREC protocol. **A**: Resampling Stage I: secondary structure is determined from chemical shift data. If strands exist all possible pairings are enumerated and 0-2 of them are randomly enforced for each structure generated using a noncontinuous fold-tree to enforce the pairing(s) (cf. text). **B:** Resampling Stages II–III: Beta-topology sampling. Low-energy decoys are analyzed to extract the more frequent beta-sheet topologies. These are subsequently enforced via a noncontinuous fold-tree. **C,D**: Resampling Stage IV: (C) Fragment resampling. Local structure is extracted from low-energy decoys and replaces the original fragment library. (D1-D3) Proto-fold resampling. (D1) Initial trajectories (blue, red, green lines) are launched from a mountain top and sample different slopes (i.e., folds) of the mountain. (D2) Different helical topologies drawn in corresponding colors illustrate possible irreversible fold-decisions made early in an annealing trajectory. (D3) The green topology (box in D2) is lowest in energy and hence the green trajectory is selected for resampling. Three new trajectories are started from an early snapshot(red cross) of the original green trajectory (green). While the resampled trajectories follow the same slope downhill, considerable freedom for exploration remains. **E**: Resampling Stages V-VI: All-atom refinement after loop-rebuilding, and core-resampling.

The individual resampling stages—explained below—are (i) beta-topology sampling with the random pairs protocol, (ii) a combination of random pairs and topology resample, (iii) topology resampling (iv) fragment resampling and proto-fold resampling (v) loop-closing and all-atom refinement and finally (vi) core-resampling, (loop-)fragment resampling and all-atom refinement (Figure 1).

### Strand–strand topology resampling (stages I–III)

As illustrated in Figure 1 the RASREC protocol first determines the strand regions from the secondary structure prediction [Figure 1(A)]. On the basis of these strand-definitions possible beta-sheet topologies are generated varying the order of strands as well as the direction (parallel and antiparallel) and register [Figure 1(B)].

To efficiently construct conformations that sample specific strand-topologies we represent the protein using a broken-chain “fold-tree”^11^, ^16^, ^17^ which is a core-feature in ROSETTA3.0.^17^ In a broken chain fold tree, changes in torsional degrees do not propagate from N- to C-terminus, but instead a directed acyclic graph is created with edges being either peptide bonds or higher order contacts (as in a beta-sheet). Peptide-plane orientations for a strand–strand contact are sampled from a library constructed from beta-sheet regions of high-resolution X-ray structures. From this strand–strand library we copy the phi, psi torsional angles of paired residues, and compute a rigid-body transform that relates the N-CA-CO planes of both residues through space. These nonconsecutive fragments from the strand–strand library are sampled during the fragment-assembly just like the usual peptide fragments (of 3 and 9 residue length). Given an input topology that defines a strand–strand pair, as well as orientation (parallel, antiparallel), and pleating (inwards, outwards) we copy suitable orientations and torsions into the fragment library for this nonlocal fold-tree edge. This generalizes the fragment-library used for ROSETTA *de-novo* fragment assembly to contain nonlocal fragments, and has been implemented in ROSETTA3.0.^17^

The fold-tree must not contain cycles, thus for each nonlocal strand–strand edge a peptide edge has to be removed somewhere else along the chain preferably in loop-regions. These chain breaks are closed in Stage V prior to all-atom refinement (cf., Section Loop-closing).

At the beginning of each folding trajectory a specific fold-tree is constructed by choosing suitable strand–strand edges and chain break positions. The choices are governed by two different strategies, called RANDOM_PAIRS and TOPOLOGY_RESAMPLE.

#### Random pairs

All residues with more than 70% sheet content in the (3mer) fragments are treated as putative beta residues. All stretches of 3 or more beta residues are considered a strand. A list of all possible pair-wise combinations of beta residues within different strands is generated with 4 entries per pair to cover all possible orientations and pleatings (see above). For each fragment-assembly trajectory up to two pairings are selected from this list with the only constraint that chosen pairings must not connect the same two strands twice.

#### Topology resample

In this mode a topology is defined by specifying exactly which strand–strand pairs are in contact and in which orientation and pleating. For a given topology with N strand–strand pairs, we select up to N pairs for enforcement. For each enforced strand–strand pair exactly one residue pair is selected to become an edge in the fold-tree. The orientation and pleating of this pair defines the allowable rigid-body orientations for this edge.

A topology is extracted from a decoy-conformation by using the DSSP definition of hydrogen bonds for the NH-CO hydrogen bonds^18^ and by combining nearby pairings with compatible pleatings and registers into “strand–strand” pairs. Given an ensemble of conformations a list of topologies is generated. A score is computed as described in the following paragraph, and only topologies whose score is at least 80% of the top-score are selected.

The scoring rewards topologies whose individual strand–strand pairs are frequently occurring in the set of decoys. Strand–strand pairs from different decoys are considered equivalent if they have the same orientation and register, and if the pleating pattern matches. Thus, two equivalent strand–strand pairs may have different sets of hydrogen-bonded residues, as long as all other parameters match up (e.g., antiparallel strands 1–10, 2–9, 3–8, and 2–9, 3–8, 4–7 are equivalent if the first strand starts with inward pleating and the second with outward pleating). Each strand pair is assigned a score *S*_sp,I_ = *N*_*i*_ (*L*_*i*_ −1+0.2max(0, CO_*i*_-20)), where *N*_*i*_ is the number of occurrence of this or equivalent strand–strand pairs, *L*_*i*_ is the number of paired residues in the strand–strand pair, and CO_*i*_ is the maximum sequence separation within this strand pair. Low contact-order strand–strand pairs are more frequent, than high contact-order. Thus the score rewards high contact order (CO_*i*_ > 20). Moreover, we prefer longer stretches (*L*_*i*_) of consistent pairings, which are more likely to be found in a region where strand–strand pairings occur natively. The total score of a topology is taken to be the sum of the scores of its strand pairs. More complex schemes to predict beta topologies based on ROSETTA full-atom energy have been explored elsewhere.^12^

#### Fragment resampling (stages III–VI)

Fragment assembly in Rosetta is a Metropolis Monte-Carlo sampling of backbone torsion angles from a diverse library of protein structure fragments. The lowest scoring conformations generated in fragment assembly trajectories are selected, and new libraries of fragments harvested from the low-scoring decoys are used for resampling in Stages III–VI [Figure 1(C)]. Fragments are derived from structures that have idealized bond-lengths and angles, and stretches of residue that contain a chain-break are excluded.

#### Proto-fold resampling (stage IV)

Models with the roughly correct overall topology can often be discriminated by ROSETTA low-resolution energy and sparse NMR restraints. However, usually these emerging structures are still of low quality in the core-region, and thus inadequate for full-atom refinement that focuses on loop-rebuilding.^15^ Inspired by ideas in Ref. 14, we intensify the conformational search around the identified—partly correct—folds by restarting from early snapshots of successful annealing trajectories.

As illustrated in Panels D1–D3 of Figure 1, the early part of a fragment assembly trajectory can be seen as a sequence of decisions on the fold, which cannot readily be corrected once the structure becomes compact in the later part of the fragment assembly trajectory. By going back to an early snapshot of a successful donor-trajectory [red cross in Figure 1(D3)] we start the sampling from a point which is poised towards a similar (low-scoring) region in fold-space. Since the selected donor-snapshots are not yet well compacted due to the short simulation time their ROSETTA energy and restraint energy is usually little informative. However, the quality of the fold-decisions encoded in these snapshots can be judged indirectly via the final snapshots of the donor-trajectories, since for these fully compacted decoys the ROSETTA energy and the restraint energies are quite informative. As indicated in Figure 1(D), at the top it is almost impossible to predict which trajectory reaches the wide-open lowlands. Nevertheless it is clear that the flank of the mountain-peak that is descended strongly determines the outcome of the trajectories. An example of such a proto-fold is the first hairpin in the WW domain, which Shaw *et al*. found generally precedes folding to the native structure.^19^

#### Loop-closing (stages V, VI)

For all proteins containing beta-sheets a nontrivial fold-tree is introduced in Stages I–III. For each enforced beta-pairing, also a nonphysical chain break somewhere in the peptide chain between the paired residues is required to avoid a cyclic fold-tree. These chain breaks must be closed to yield physically realistic models. Moreover, a chain break that cannot be closed is a strong criterion to reject a structure. Three mechanisms for closure exist which are combined. First, a chain break penalty is computed during the fragment assembly and its contribution to the overall energy increases each sampling cycle. Second, chain-breaks are removed by idealization of the chain-break residue, and third, by explicit loop-rebuilding. Two chain break penalty functions are used: the first depends only on distance, the second, also on the orientation of consecutive peptide planes; both penalties are linear (rather than quadratic) in the deviation from perfect closure.

All RASREC trajectories apply the chain break energies, but explicit removal of the chain breaks is postponed until all-atom sampling commences. At this point, structures are often already well folded and the chain-break energy has had time to select for close to ideal loops. To remove the chain breaks entirely, an application of the ROSETTA idealization protocol is attempted; if unsuccessful loop-rebuilding is attempted instead. Chainbreak closure by idealization and loop rebuilding are described in detail in the following two sections.

##### Chainbreak removal by idealization

The idealization protocol generates strong positional restraints to bias towards the current conformation and then replaces the bond-lengths and angles of the chain-break residue with ideal values. The idealization introduces a perturbation of the backbone that can result in large amplitude motion downstream from the idealized chain break due to lever-effects along the peptide backbone. Subsequent minimization in torsion space under the influence of the positional restraints often succeeds in decreasing the downstream motion to <0.1 Å RMSD. However, if the chain-break is far from ideal, idealization will perturb the structure too much to recover the original conformation by minimization and the idealization-attempt is considered a failure.

##### Chainbreak removal by loop-rebuilding

Loops that contain the chainbreak residue are rebuilt via fragment assembly until ideal closure without large-amplitude downstream effects can be achieved. Rebuild-windows are selected from the region between adjacent fold-tree nodes. First smaller windows are attempted and then progressively larger regions are rebuilt until the maximal allowable region (between fold-tree nodes) has been rebuild. For short windows (less than 10 residues) 100 monte-carlo steps of fragment assembly using only the chain break energy terms (unscored loops) are followed by cyclic coordinate descent (CCD) loop closure.^20^ For larger windows (more than 10 residues) a simplified energy function with vdW, env, pair, and chain break terms with equal weights is used (scored loops). Loop-conformations whose vdW- and chain break energies are at most 0.5 energy units higher than the starting conformation's are considered good. When more than 80 unscored or more than 5 scored good loops are generated all loops are rescored with the standard low-resolution energy function and the best-scoring loop is selected. In this selection the initial conformation that has not been changed by fragment assembly gets an equal opportunity to be selected. In this fashion backbone regions across all chain breaks are rebuilt and subsequently the whole chain is idealized.

#### Use of NOESY restraints in RASREC (all stages)

Fragment assembly in the context of a set of distance restraints (such as NOEs) is difficult due to the ruggedness of the energy landscape and the large amplitude of the changes produced by individual moves.^3^ To avoid frustration of the sampling due to long-range restraints, restraints are switched on in order of their sequence separation.^3^ The sequence separation is computed according to the chosen fold-tree of a given trajectory: For a pair of residues the shortest path is computed counting each peptide bond and a connection via a strand–strand edge of a nontrivial fold-tree as one. Thus, the chain break penalty is switched on according to the residue's separation in the fold-tree.

### The Archive framework

An “Archive”-framework was developed to facilitate development and implementation of iterative refinement protocols. The infrastructure allows distribution of RASREC-Rosetta as a fully automatic and platform independent application and thus significantly lowers the entry-barriers for new users.

Iterative structure determination protocols have been explored in our lab and by others.^11^, ^14^, ^15^, ^21^ They are often implemented on a scripting level and involve considerable manual interaction. Because of the manual labor involved (1) the work is less reproducible, which hinders rigorous method development and (2) considerable barriers need to be overcome for adoption by others. Moreover, fluctuating processor usage is an intrinsic problems of iterative structural sampling that causes inefficiencies and bottlenecks.

Parallelization of the method without significant loss in performance is challenging. A large number of CPUs are used for conformational space exploration, whereas a single CPU is used for data collection and analysis between exploration stages. Processes in the exploration phase can vary drastically in length—for the Rosetta *de novo* protocol this can be as much as 50–200% from the average and hence many CPUs may idle while waiting for completion of the slowest job (idle CPUs can be used by other tasks via a queuing system, but this can result in long wait times in the queue until the resources become available again). We first explored the use of the DAG-manager from the CONDOR queuing system^22^ to integrate our scripts with the queuing system of our in-house cluster. This allowed for a largely automatic running of the protocol, but requeuing indeed caused bottle-necks. A further drawback of this solution was that a new implementation was required for each queuing system. Inparticular, when using public computer centers one often has no control over the choice of the queuing system. Finally, with increasing run sizes we started to have issues with file-IO contention due to large amount of file-opening and closing, since every independent process independently writes its results to disk.

#### MPI based job-distribution

To overcome these issues we decided to implement job distribution using MPI within the ROSETTA3.0 framework^17^ such that a single large-scale job can be issued on a shared computer cluster that continuously utilizes 100–2048 cores without idling. Technically the framework scales well to even larger numbers of cores, but in the context of the RASREC protocol we find 500–2048 cores close to optimal. Within the framework, different processors can employ different ROSETTA structural sampling protocols—*de-novo* folding, loop-modeling, low- and high-resolution refinement. With 600–6000 s per trajectory on the BlueGene computer, 2048 processes generate up to four structures a second, and care had to be taken such that analysis and job-distribution do not cause bottlenecks.

**Figure 2:**
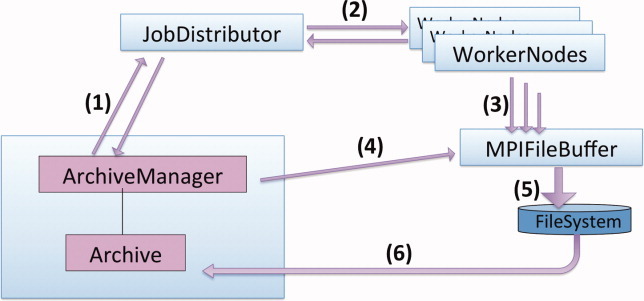
Illustration of the Archive-framework for job-control and iterative structural sampling. Each blue box is a distinct process and arrows (1–4) indicate Message Passing Interface (MPI) based communication. Arrows (5–6) are file-IO operations. The individual communications are (1) (up) queuing of new batches (down) notification of finished results and underflow of the queue (2) (right) start jobs (left) report success/failure (3) write to file (4) lock/release files before reading with (6) to avoid competition with writing (5). The architecture is modular and the RASREC protocol is implemented by specializing the base-class “Archive”. The MPI-based JobDistributor is now also used in non-iterative contexts within ROSETTA to efficiently execute a number of tasks within a single job. [Color figure can be viewed in the online issue, which is available at wileyonlinelibrary.com.]

The software design is illustrated in Figure 2. Three processes are dedicated to organizational tasks; these are single-point File-IO, job distribution, and structural analysis, respectively. The framework allows analysis and issuing of resampling tasks in parallel to the sampling of trajectories.

The single-point File-IO (*MPIFileBuffer*) exports virtual files to individual worker processes such that any existing rosetta protocol that uses the ROSETTA3.0 interface for structure IO (silent-IO)^17^ will continue to work properly. Silent-IO frames of individual worker processes are collected from the *MPIFileBuffer* process and written to a single common file while avoiding the scrambling of lines from different frames. No code changes on the protocol level were required thus allowing the use of any Rosetta protocol within this framework.

The *Jobdistributor* maintains lists of batches (one per iteration) and jobs. Worker processes ask for a job, perform the calculation and write the result to file. Subsequently, they notify the job-distributor about the job's conclusion (e.g., SUCCESS, FAILURE) and inquire about new jobs. If the job-queue is empty the Jobdistributor will prompt the archive to generate flags and options for a new batch.

The *ArchiveManager* maintains the pool of structures. It is notified by the Jobdistributor if more than 100 new decoys are available for reading from one of the current batches. The structures are subsequently read and accepted into the pool based on predefined scoring criteria. The ArchiveManager increments to the next resampling stage when the acceptance ratio drops below 10%. When prompted by the Jobdistributor new batches are generated using the resampling strategy of the current stage.

More implementation details are given in Supporting Information Section 2.1.

### Benchmark

The RASREC protocol was run on 11 targets with 120–200 residues of distinct folds with all-alpha, alpha/beta, and all-beta topologies (Table 1). For all targets initial fragments were selected using chemical shift data obtained from the BMRB.^1^ Additional restraints such as N–H RDCs (RDC) or backbone H^N^–H^N^ NOE restraints (NOE) were used as indicated in Table 1. If available in the BMRB experimental RDC restraints were used, otherwise RDC restraints were simulated using the reference structure (details in Supporting Information Section 2.2). NOE restraint sets were either obtained by filtering experimental (obtained from BMRB) sets of NOE restraints (labeled Exp.) or by generating distance restraints for contacting backbone amide protons in the reference structure (labeled Sim) (details in Supporting Information Section 2.2). For 10 targets two RASREC runs were performed one with more and one with less data (CS vs CS+RDC; CS+NOE vs CS+RDC+NOE; or CS vs CS+RDC+NOE; Table I). As a control each run was repeated using the standard CS-Rosetta protocol as described in Ref. 1 using exactly the same input data. For both protocols 24 h computer time was allocated. RASREC stops automatically when converged and thus did not always use its full time-allocation.

**I:** Benchmark Set

				Experimental data used				Residues used for RMSD	
					
	Target	Size	Fold	CS (BMRB)	RDCs	NOEs	#NOE	Less data	More data
1	2jyx	124	α/β	15603	Exp(+)	Exp	16	1–121	1–121
2	1f21	142	α/β	4012	Sim(+)	–	0	1–70, 79, 96–120	1–70, 79, 96–120
3	2k1s	143	α/β	15683	Exp(+)	–	0	1–143	1–143
4	2kd7	150	β	16107	Exp(+)	Exp	21	1–150	1–150
5	1i1b	151	β	434/1061	Sim(+)	Sim(+)	33	1–151	1–151
6	1i1b_2	151	β	434/1061	Sim(+)	Sim	84	1–151	1–151
7	5pnt	157	α/β	5350	Sim(+)	–	0	4–47, 68–157	4–47, 68–157
8	1s0p	160	α	5393	Sim(+)	–	0	1–160	1–160
9	2k5u	166	α/β	15626	Exp(+)	Exp(+)	16	1–166	1–166
10	2z2i	179	α/β	7055	Sim(+)	Sim	27	1–13, 23–112, 118–139, 162–179	1–179
11	2jzc	201	α/β	15617	Exp(+)	Exp(+)	52	–	5–70, 81–139, 151–180

^a^Experimental chemical shift data was used in all cases to pick fragments.^1^ For RDC and NOE restraints, we used experimental data where available (Exp.) otherwise data was simulated using the native conformation (Sim.). The “(+)” marks data that was used only in the run with more data, (right side of Figure 4). Additional details on restraint generation and the used input files for RDC and NOE restraints are given in Supporting Information Section 2.2., The “–” marks structure calculations that have not been carried out.

As a quality measure for the coordinate accuracy of a structure given a reference structure we compute RMSD and GDTMM on the CA atoms. The latter measure adopts a value between 0 and 1 for random and perfect coordinates, respectively. The GDT-MM (details described in Ref. 23), a variant of the GDT_TS^24^, computes the mean alignment coverage at different RMSD thresholds and ranges from 0 (random similarity) to 1 (perfect similarity).

## RESULTS

**Figure 3:**
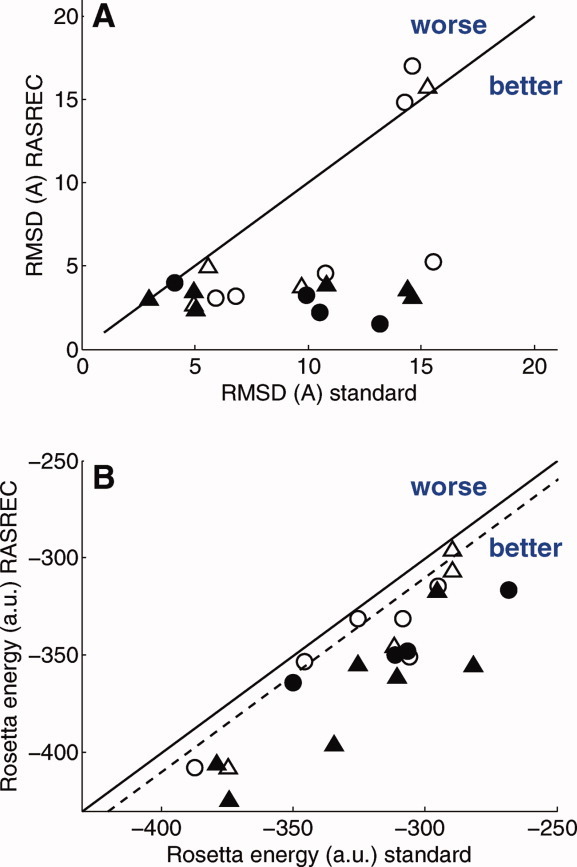
Comparison of RASREC with standard CS-Rosetta protocol. **A**: Median RMSD and (**B**) the Rosetta all-atom energy of the 10 lowest energy models for both protocols obtained using the same restraint data. The symbols indicate the data used beyond the chemical shifts. Open circles: CS only; filled: +RDC; triangles: +NOE; filled-triangles: +RDC +NOE. The dashed line marks an energy difference of 10 units which corresponds roughly to 6 kcal/mol.^25^ [Color figure can be viewed in the online issue, which is available at wileyonlinelibrary.com.]

The RASREC-protocol and standard CS-Rosetta protocols were applied to 11 proteins between 15 and 25 kDa size using different sets of sparse NMR restraints as described in the Benchmark section. As shown in Figure 3, over the 19 protein benchmark set, the RASREC protocol improved (over standard CS-Rosetta) coordinate accuracy in 13 cases and Rosetta all atom energy in 16 cases. For most targets, RASREC pushed the accuracy below 6A where the topology is well defined. For three targets neither RASREC nor CS-Rosetta yielded converged solutions when no RDC data is used (open symbols), but all three problematic targets are solved (below 5A) using RASREC with additional RDC data (filled symbols).

**Figure 4:**
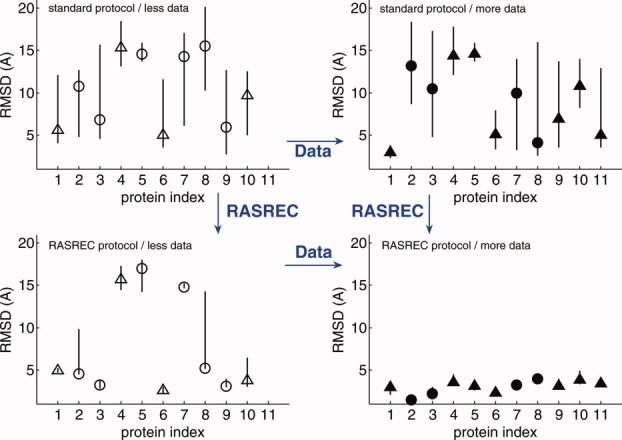
Dissecting the influence of methodical improvements (RASREC) versus additional data on improved model generation. For each protein (numbered 1–11) the median, highest and lowest RMSD of the 10 lowest energy models are shown with symbols for the median, and lines indicating the low-high interval. Results for standard CS-Rosetta and iterative CS-Rosetta are shown in the upper and lower row, respectively. In all cases, more restraint data has been used for the runs plotted on the right, less for runs on the left. For more detail refer to the symbols: open circles: CS only; filled: +RDC; triangles: +NOE; filled-triangles: +RDC +NOE. [Color figure can be viewed in the online issue, which is available at wileyonlinelibrary.com.]

Both additional restraint data and application of the RASREC protocol improve the accuracy and precision of final structures. Since targets have been run with and without RASREC and with more or less data, the influence of protocol and data can be dissected separately. As shown in Figure 4, improved models are obtained when either more data are used or the RASREC protocol is used. The overall best results are obtained (lower right corner) using RASREC and additional data. The standard CS-Rosetta protocol benefits much less from additional data than RASREC. Adding restraint data to the standard CS-Rosetta protocol yields only one accurate and precise solution (target 1, the smallest protein in the benchmark). In contrast, for RASREC the additional data not only results in convergence for three targets, but also yields significant improvements in accuracy for all targets that already converge to meaningful structures with less data. These results demonstrate that the determination of larger protein structures described in Ref. 10 required the RASREC protocol.

**Figure 5:**
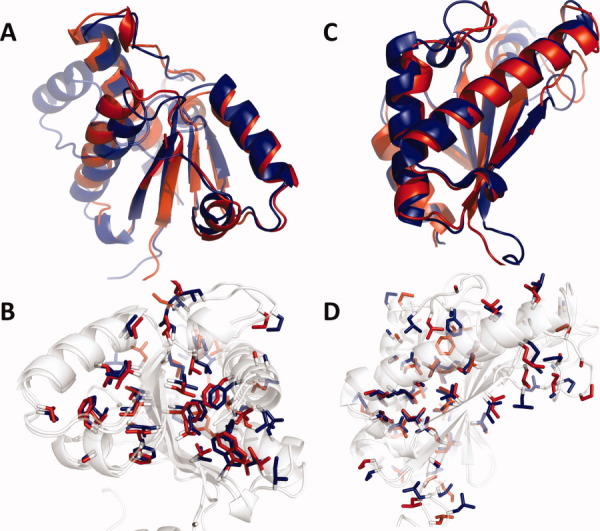
Final RASREC structures (red) for 5pnt (**A,B**) and 2k1s (**C,D**) superimposed with the independently determined reference structures (blue). (B,D) show nonpolar side-chains in stick representation. For 5pnt unconverged residues 48–67 are not shown in; this residue range is shown half-transparent for the X-ray structure (A; upper left helix). [Color figure can be viewed in the online issue, which is available at wileyonlinelibrary.com.]

**Figure 6:**
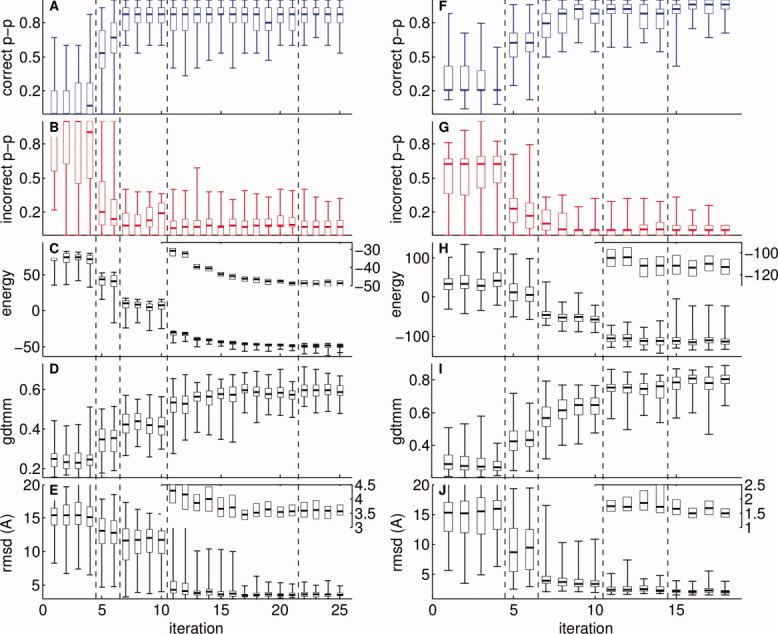
Characterization of the resampling process for targets 5pnt (**A–E**) and 2k1s(**F–J**). For each generation of decoys (*x*-axis), parameter distributions of (correct and incorrect beta pairings (p-p), energy, GDTMM and RMSD, respectively) are shown for the 5% lowest energy decoys as box-and-whisker plots (the lower and upper quartile of the distribution are shown as box with a horizontal line at the median, and extreme values as whiskers). Vertical dashed lines mark the transition to the next resampling stage (I–V; VI not shown for clarity). Insets, where present, are blow-ups of the same data, but without the whiskers for clarity. (A,B,F,G) Correct and incorrect beta pairings (p-p) are evaluated against the native structure. (E+J) RMSD is calculated on the converged residues: 4–47, 68–157, and 7–38, 46–114, 131–140 for 5pnt and 2k1s, respectively. [Color figure can be viewed in the online issue, which is available at wileyonlinelibrary.com.]

It is instructive to consider the performance of the RASREC protocol on two of the test cases—5pnt and 2k1s—in more detail. The final converged structure for these targets is shown in Figure 5 and is based on backbone chemical-shift and RDC data. Figure 6 shows distributions of structural parameters for each iteration. The energy (Row 3) drops immediately and substantially when the protocol switches to the next resolution stage (dashed lines) suggesting that the stage-dependent feature resampling indeed well matches the resolution of that stage.

After iteration 7 (8) for 5pnt (2k1s) a near perfect beta-topology is achieved (Figure 6, row 1+2). The resampling Stage II (iteration 5+6) has a strong impact on improving the beta-topology. In this stage, beta-sheet topologies of archived structures are used to seed the new trajectories for the first time. In Stage II random beta-pairings are still introduced along with the harvested topologies, whereas in Stage III (iterations 7–10) existing topologies are consolidated. The consolidation leads to further significant improvement in energy (Row 3), GDTMM (Row 4), and RMSD (Row 5).

At iteration 11 a further significant improvement of accuracy and energy is achieved due to fragment resampling and proto-fold resampling (Stage IV). For 2k1s switching to all-atom mode in iteration 15 yields a further significant improvement in GDTMM and RMSD, with final structures around 1.5 Å RMSD. For 5pnt the all-atom mode yields no measurable improvement in backbone RMSD.

**Figure 7:**
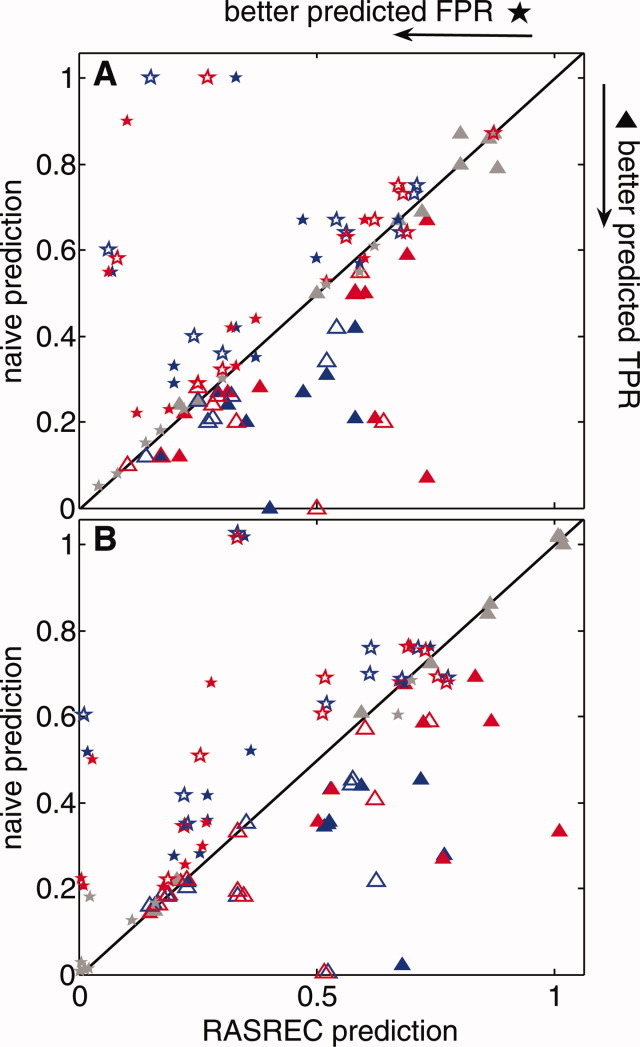
Characterization of the beta-topology predictor used for topology resampling in Stages II and III. The plots compare the true positive rate (TPR: ▴) and the false positive rate (FPR: ★) for predictions of the individual beta pairings (Panel A) and of the consolidated strand–strand pairing (Panel B). The predictors are derived from the archived 500 low-energy decoys after Stage I (blue), Stage II (red), and Stage III (gray), respectively. The *x*-axis shows FPR and TPR of the RASREC predictor as described in Section Topology Resampling (labeled: RASREC). The *y*-axis shows FPR and TPR one would obtain by simply resampling all pairings found in the 500 low-energy decoys according to their frequency (labeled: naive). Closed and opened faced symbols reflect runs with more or less data (c.f., Figs. 3 and 4). [Color figure can be viewed in the online issue, which is available at wileyonlinelibrary.com.]

Figure 6 shows a dramatic improvement of the beta-topology in resampling Stages II and III. This improvement is based on the ability to predict the beta-topology by accumulating compatible pairings as explained in Methods. Indeed, as shown in Figure 7, the RASREC predictor is significantly more accurate than predictions based solely on the raw frequencies of pairings in the low-energy decoys. In all cases, the predictor shows a higher true positive rate (TPR) and a smaller false positive rate (FPR). After resampling Stage III the predicted pairing-frequencies are fully reflected in the archived decoys, and no further improvement can be expected by the predictor (gray symbols in Figure 7).

**Figure 8:**
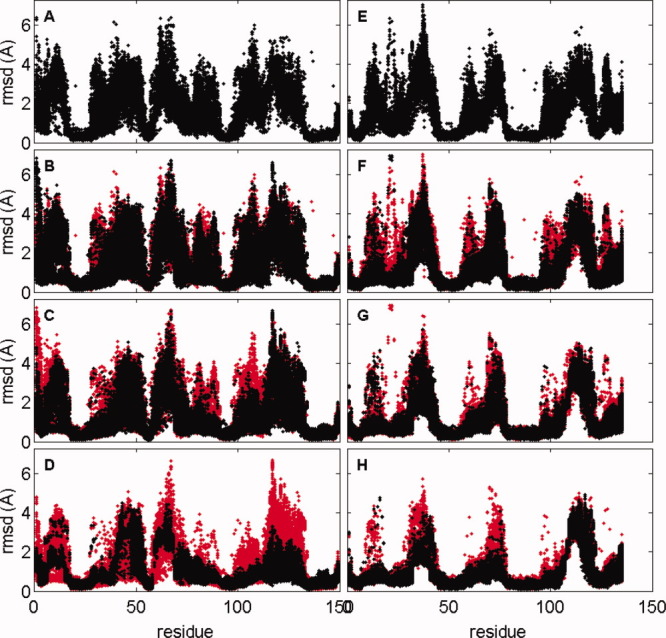
Improvement in local fragment quality with successive RASREC stages. Fragment quality for targets 5pnt (**A–D**) and 2k1s (**E–H**). For each 9mer fragment the RMSD to the corresponding local structure of the native protein is computed. A–E: 200 chemical shift selected fragments. (B–D, F–H) fragments harvested from structure archive after resample Stages II, III, and IV, respectively. For Stages II, III, and IV, the RMSDs of the prestage fragments are plotted in red to facilitate comparison with the RMSDs of post-stage fragments (shown in black). [Color figure can be viewed in the online issue, which is available at wileyonlinelibrary.com.]

In resampling Stage IV, fragment resampling is used to increase the frequency of near-native structures. To check the quality of fragments, RMSDs of 9mer fragments are computed against the corresponding stretches of backbone in the native structure. Figure 8(A,E) shows the quality of the initial fragment sets for targets 5pnt and 2k1s, respectively, there is high accuracy and precision for helical regions but considerable diversity otherwise. The subsequent rows in Figure 8(B–D, F–H) show the fragment quality for fragments obtained by chopping up the 500 decoys that are maintained as a structure pool by RASREC. The fragments shown in C and G are used for resampling in iteration 11. The diversity is much reduced without reducing the accuracy of the best fragments, and in some regions significantly more accurate fragments are found. At the end of resampling Stage IV [Figure 8(D,H)] diversity is further reduced. For 2k1s, residues 1–143, and 5pnt, residues 75–150, fragments resemble a tight and accurate structural ensemble. However, for 5pnt residues 5–15 and 40–75 convergence has also removed some of the high-quality fragments.

Although no new backbone torsion angles are introduced in the fragment assembly stages, new fragments are generated by combination of adjacent torsional angles from different source fragments. This effect can be observed in Figure 8. Thus, repeated fragment assembly efficiently removes inaccurate local backbone structure and even creates new, more accurate, local structure. By reusing the harvested rather than the initial fragment library these advances in the structure calculation are passed on to subsequent trajectories.

## DISCUSSION

To overcome the size limitations of sparse data driven structure determination methods one can employ more sophisticated algorithms, like RASREC, or use additional experiments to obtain further restraint data. The results presented here suggest that our recent breakthrough in solving larger structures^10^ was driven by algorithmic improvements more than by incorporation of additional restraint data. The standard protocol fails to converge to the correct fold for all 10 proteins in the benchmark (all 10 lowest energy structures with RMSD ≤ 5 Å; proteins 1–10). Of these failures four are recovered using the new protocol (proteins 1,3,6,9) and the remaining six by employing additional data and the new protocol together (proteins 2,4,5,7,8,10). In contrast, incorporating additional data into the standard protocol recovers only 1 of the 10 failed proteins (protein 1). Overall, RASREC improves coordinate accuracy significantly in 14 and the ROSETTA energy in 18 of 21 benchmark cases.

RASREC uses discontinuous chain folding and the idea of resampling promising strand pairings and local structural features introduced by Bradley *et al*.^11^ and Blum *et al*.,^12^ but goes beyond these previous approaches by iterating resampling through many rounds of model generation, analysis of promising features, and recombination. Like Brunette *et al*.,^13^, ^14^ RASREC identifies early stage models that lead to very low energy final models, and resamples from these, but in the context of the feature identification and recombination. As in Qian *et al*.,^15^ RASREC uses iteration to progressively improve a model population while ensuring that the appropriate level of diversity is maintained, but rather than just rebuilding single regions RASREC builds structures starting from scratch and uses feature analysis to generate promising recombinants. RASREC goes far beyond all the previous approaches by using resolution-adapted strategies at each stage of the structure generation process from an extended chain to a tightly packed structure.

## CONCLUSIONS

By developing an iterative sampling protocol that enriches native-like features by resolution-adapted feature recombination (RASREC) we have overcome typical size limitations in Rosetta *de-novo* structure generation. Our results show that a strong synergy exists between sparse (NMR) restraint data and the ROSETTA methodology. For structures >15 kDa, the recombinant resampling method proves much more proficient in exploiting this synergy than the standard CS-Rosetta protocol. These results indicate the importance of continued development of enhanced sampling methods.

## References

[1] Shen Y, Lange OF, Delaglio F, Rossi P, Aramini JM, Liu G, Eletsky A, Wu Y, Singarapu KK, Lemak A, Ignatchenko A, Arrowsmith CH, Szyperski T, Montelione GT, Baker D, Bax A (2008). Consistent blind protein structure generation from NMR chemical shift data. Proc Natl Acad Sci USA.

[2] Hirst SJ, Alexander N, Mchaourab HS, Meiler J (2011). RosettaEPR: An integrated tool for protein structure determination from sparse EPR data. J Struct Biol.

[3] Rohl CA, Strauss CEM, Misura KMS, Baker D (2004). Protein structure prediction using Rosetta. Methods in enzymology.

[4] Bowers P, Strauss C, Baker D (2000). De novo protein structure determination using sparse NMR data. J Biomol NMR.

[5] Kryshtafovych A, Venclovas Č, Fidelis K, Moult J (2005). Progress over the first decade of CASP experiments. Proteins: Struct Funct Bioinform.

[6] Bonneau R, Ruczinski I, Tsai J, Baker D (2002). Contact order and ab initio protein structure prediction. Protein Sci.

[7] Cavalli A, Salvatella X, Dobson CM, Vendruscolo M (2007). Protein structure determination from NMR chemical shifts. Proc Natl Acad Sci USA.

[8] Plaxco KW, Simons KT, Baker D (1998). Contact order, transition state placement and the refolding rates of single domain proteins. J Mol Biol.

[9] Raman S, Vernon R, Thompson J, Tyka M, Sadreyev R, Pei J, Kim D, Kellogg E, DiMaio F, Lange OF, Kinch L, Sheffler W, Kim B-H, Das R, Grishin NV, Baker D (2009). Structure prediction for CASP8 with all-atom refinement using Rosetta. Proteins: Struct Funct Bioinform.

[10] Raman S, Lange OF, Rossi P, Tyka M, Wang X, Aramini JM, Liu G, Ramelot TA, Eletsky A, Szyperski T, Kennedy MA, Prestegard J, Montelione GT, Baker D (2010). NMR structure determination for larger proteins using backbone-only data. Science.

[11] Bradley P, Baker D (2006). Improved beta-protein structure prediction by multilevel optimization of nonlocal strand pairings and local backbone conformation. Proteins: Struct Funct Bioinform.

[12] Blum B, Jordan MI, Baker D (2010). Feature space resampling for protein conformational search. Proteins: Struct Funct Bioinform.

[13] Brunette TJ, Brock O (2005). Improving protein structure prediction with model-based search. Bioinformatics.

[14] Brunette TJ, Brock O (2008). Guiding conformation space search with an all-atom energy potential. Proteins: Struct Funct Bioinform.

[15] Qian B, Raman S, Das R, Bradley P, McCoy AJ, Read RJ, Baker D (2007). High-resolution structure prediction and the crystallographic phase problem. Nature.

[16] Karplus K, Karchin R, Draper J, Casper J, Mandel-Gutfreund Y, Diekhans M, Hughey R (2003). Combining local-structure, fold- recognition, and new fold methods for protein structure prediction. Proteins: Struct Funct Bioinform.

[17] Leaver-Fay A, Tyka M, Lewis SM, Lange OF, Thompson J, Jacak R, Kaufman K, Renfrew PD, Smith CA, Sheffler W, Davis IW, Cooper S, Treuille A, Mandell DJ, Richter F, Ban Y-EA, Fleishman SJ, Corn JE, Kim DE, Lyskov S, Berrondo M, Mentzer S, Popović Z, Havranek JJ, Karanicolas J, Das R, Meiler J, Kortemme T, Gray JJ, Kuhlman B, Baker D, Bradley P (2011). ROSETTA3: an object-oriented software suite for the simulation and design of macromolecules. Methods Enzymol.

[18] Kabsch W, Sander C (1983). Dictionary of protein secondary structure: pattern recognition of hydrogen-bonded and geometrical features. Biopolymers.

[19] Shaw DE, Maragakis P, Lindorff-Larsen K, Piana S, Dror RO, Eastwood MP, Bank JA, Jumper JM, Salmon JK, Shan Y, Wriggers W (2010). Atomic-level characterization of the structural dynamics of proteins. Science.

[20] Canutescu AA, Dunbrack RL (2003). Cyclic coordinate descent: A robotics algorithm for protein loop closure. Protein Sci.

[21] Perrakis A, Morris R, Lamzin VS (1999). Automated protein model building combined with iterative structure refinement. Nat Struct Biol.

[22] Condor Team UOW-M http://www.cs.wisc.edu/condor/manual/v7.0.

[23] Thompson J, Baker D (2011). Incorporation of evolutionary information into Rosetta comparative modeling. Proteins: Struct Funct Bioinform.

[24] Zemla A, Venclovas C, Moult J, Fidelis K (1999). Processing and analysis of CASP3 protein structure predictions. Proteins: Struct Funct Bioinform.

[25] Kellogg EH, Leaver-Fay A, Baker D (2010). Role of conformational sampling in computing mutation-induced changes in protein structure and stability. Proteins: Struct Funct Bioinform.

