# Supplementary material for: Resolution-adapted recombination of structural features significantly improves sampling in restraint-guided structure calculation
Source: Proteins. 2011 Nov 9;80(3):884–95. doi: 10.1002/prot.23245 (PMC3310173; doi:10.1002/prot.23245)
Supplement: Supplementary file 1 [file prot0080-0884-SD1.pdf]

Supplemental Material to: Resolution-adapted recombination of structural features significantly improves sampling in restraint guided structure calculation.

Oliver F. Lange and David Baker

1. Table of Contents

2. SUPPLEMENTARY METHODS 1
2.1. MPI-FRAMEWORK 1
2.1.1. Overview 1
2.1.2. FileBufJobDistributor 2
2.1.3. ArchiveJobDistributor 2
2.1.4. FileBuf 2
2.1.5. MasterProcess 3
2.2. NMR RESTRAINTS FOR BENCHMARK 5
2.2.1. Simulated RDC restraints 5
2.2.2. NOE Restraints 5
2.2.3. RDC Restraint Input Files 6
2.2.4. NOE Restraint Input Files 18

2. Supplementary Methods

2.1. MPI-Framework

2.1.1. Overview

The `JobDistributor` maintains a job-list and applies an instance of `Mover` to each job. In single processor-mode the JobDistributor will look in the designated output file which job has already been processed. Originally the output file was scanned before each new job is processed, however, this can lead to file system contention. A safer version looks for existing output only at start-up, but this renders workload management between multiple processes impossible. Moreover, it is not possible to write to a single output file from multiple processes due to unhandled race conditions.

The Message Passing Interface (MPI) allows platform independent communication between processes and has been used for dynamic workload management and control of race conditions in resource allocation (i.e., output file). Previous MPI-specializations of the `JobDistributor`, the `MPIWorkPoolJobDistributor` and `MPIWorkPartitionJobDistributor`, block after a job succeeded such that file-IO race conditions are avoided. However, this implies a long-wait

time during which file-IO takes place and does not avoid race-conditions for protocols that want to write to more than the official output file. However, this is the case for the conformational-slope protocol, which requires writing of intermediate snapshots. The `MPIFileBufJobDistributor` overcomes these issues by separating IO from workload management (Figure 2; main text). Thus, in *batch* mode two processes are dedicated to job distribution and file-IO. In *iterative* mode an additional *master* process is introduced that issues new batches to the batch-queue.

2.1.2. *FileBufJobDistributor*

The `jd2::MPIFileBufJobDistributor` is inherited from `jd2::JobDistributor` and overrides the virtual functions `go, get_new_job_id, new_batch, and job_succeeded.` Further, it provides new hooks, to allow further speciazilation, most importantly `process_message.`

Incoming MPI messages are processed in `process_message()`, messages are NEW_JOB_ID, BAD_INPUT, and JOB_SUCCESS. Slave jobs ask for a new job- and batch-ID. The jobID is an index into the job-list, the batchID identifies the job-list to be used. If no more jobs are available the slave receives a NULL job-ID that causes him to spin down.

Batches are a set of jobs that are generated by a common set of flags. Usually all batches are known from the start. They can be given to the command-line by `run:batches flags1 flags2 flags3` etc.

2.1.3. *ArchiveJobDistributor*

In *iterative* mode batches are not known at start time, but are generated based on structural analysis of incoming decoys. To allow dynamic additions to the batch-queue the `MpiFileBufJobDistributor` is extended to the `MPIArchiveJobDistributor.` A BATCH_SYNC message is issued by a slave process that received a job with batchID larger than the slave's `nr_batches()`. Subsequently, `batchID-nr_batches()` ADD_BATCH messages are sent to update the slaves batch-list. Newly generated batches are added to the master batch queue by the JobDistributor upon receipt of an ADD_BATCH message from the `ArchiveManager`-process. Old but still unprocessed batches can be obsoleted from the ArchiveManager by sending a CANCEL_BATCH message to the Jobdistributor. No more jobs from a cancelled batch are issued and incoming decoys are not passed on to the ArchiveManager. This is mainly used when switching to a new resampling stage.

When the batch-queue is empty the ArchiveManager is prompted for additional work before taking the default action of spinning down. To this end, the virtual `batch_underflow()` method has been `overridden to send a QUEUE_EMPTY message to the ArchiveManager.`

2.1.4. *FileBuf*

All work processes that work on the same batch write their results into the same set of files. Parallelization is now compeltely hidden from work-processes and setup, i.e., no process-dependent filenames, such as result_0001, result_0002, etc, are necessary. File output in Rosetta is generally handled via the `ozstream` class, as a requirement to be able to build the the Rosetta@home executable. To allow parallel output for all Rosetta protocols without changes in protocol source code, we simply replace the underlying `streambuf` of the ozstream object. The replacement with mpi_stream_buf (mpistream.hh) is triggered by calling the static method `enable_MPI_reroute()` (done in `::go` method of `MPIXXXJobDistributor`).

File-operations such as open, close, write and flush are now related to the dedicated IO-process that runs an intance of `MPIFileBuffer.` Each file opened by a slave process creates a corresponding buffer in `MPIFileBuffer`, whose contents is only written to disk after `flush()` or `close()` is called on the `mpi_stream_buf.` Typical behavior for ROSETTA protocols is to open and close a file for each decoy generated. Thus, srambeling of lines between, e.g., silent-IO frames coming from different processes, is avoided by the unique slave-process dependent buffers.

For communication between mpi_stream_buf and MPIFileBuffer the following messages have been created: MPI_STREAM_OPEN, MPI_STREAM_SEND, MPI_STREAM_CLOSE, and MPI_STREAM_FLUSH. Messages MPI_BLOCK_FILE and MPI_RELEASE_FILE are used in *iterative* mode to avoid race-conditions when decoys are read by the master process (ArchiveManager) while slave-processes are writing into the same file. Due to the buffered output slave-process continue writing despite the blocking, however, finished frames (flush/close) will not be written from the internal buffer to to the disk.

2.1.5. MasterProcess

a. ArchiveManager

An instance of `ArchiveManager` is the top-level controller in *iterative* mode. This class encapsulates all basic infrastructure work that is common to any iterative protocol while high-level details, i.e., decision making, is handed over to an instance of an `ArchiveBase`. The most important interface methods of `ArchiveBase` called by the `ArchiveManager` are `generate_batches()` and `read_structures()`. The former is called when the QUEUE_EMPTY message has been received from the Jobdistributor (see above) and the latter method is called when new decoys are available in one of the batches output files.

When the `ArchiveManager` receives JOB_COMPLETION messages issued by the job-distributor it reads the respective output file and compares decoys-tag to figure out which decoys in the file have not been read into the archive. The decoys with new tags are read and forwarded to the the Archive using `read_structures()`. Additionally, the Manager oversees storing of recovery information and restart, the cancelling of batches and transmission of a stop-signal to the Jobdistributor if no new batches are generated.

For each new batch a directory batch_nnnnn is created where nnnn represents the number of the batch. This directory contains flags and setup files, and takes all output-files. The directory contains a file BATCH_INFO which is maintained by the ArchiveManager to store status information regarding that batch in case that a run has to be restarted. The class `Batch` provides filenames for the typical files (flags, setup-files, output-decoys) and represents the BATCH_INFO information in memory.

b. The ArchiveBase Hierarchy

b.i. ArchiveBase

The `ArchiveBase` provides an interface for a general controller of iterative protocols that maintain a pool of structures as common feature. It provides methods `read_structures()` to add fresh decoys to the archive, and `generate_batches()` to issue new (resampling) work. Moreover, the interface provides hooks to safe and restore the archive content and status.

b.ii. EvaluatedArchive

For RASREC, the decision which structures to keep, is based on a weighted sum of scores. This decision is handled by the `EvaluatedArchive` subclass. Any iterative protocol that is based on a pool of structures that are selected via a weighted some of energies and other metrics would be derived from this class. To give a negative example, a Pareto selection scheme, for instance, would have to implement a different selection scheme, and would not be derived from this class.

A clean modular design would compute all energies and metrics of incoming decoys when they arrive. However, on the BG/P system with relatively slow single processors and 2048 slave processors rescoring of incoming structures was too expensive. Benchmarking revealed that not the rescoring, as one would expect, but the generation of poses out of silent-file frames was causing the bottleneck. To avoid this problem a non-local evaluation is toggled by `set_evaluate_local( false )` such that finished decoys have to be evaluated from the generated slave-job before submitted to file. The compound-score is stored as `score_final` in the silent-file. Obviously, with decoys coming from different batches care has to be taken that the values in `score_final` are consistent. This is really against good software engineering practice and caused all the commonly expected problems. Much later we found a way to make pose-generation out of silent-frames much faster (as implemented in current svn), such that the non-local evaluation might be obsolete. This design-decision is worth revisiting before further method-development.

Individual metrics or energies can be computed from each incoming decoy using instances of `evaluation::PoseEvaluator` and an instance of `ScoreFunction`. The weighted sum is cached as _archive_select_score_ in the silent-struct of each archived decoy. Only when weights are changed or score-terms added, these values get re-evaluated. All necessary book-keeping for the chaching mechanism is handled by EvaluatedArchive.

b.iii. IterativeBase (RASREC_Base)

The logic of the RASREC protocol is implemented in classes IterativeCentroid (stages I-IV) and IterativeFullatom(stages V-VI), both classes are derived from IterativeBase. A wrapper class called IterativeAbrelax bundles IterativeCentroid and IterativeFullatom into a single object. Most of the functionality is implemented in the base class.

The class overloads `generate_batch()` to issue new batches based on its pool of decoys (accessed with decoys()).

Methods to setup input files for the batches are called gen_XXX and are invoked in stage-dependent manner from generate_batch(). These methods are gen_enumerate_pairings(I-II), gen_resample_topologies(II-III), gen_resample_stage2(IV), gen_resample_fragments(IV-VI), gen_cen2fullatom(V), gen_cen2fullatom_non_pool_decoys(V) in IterativeBase and gen_resample_core(VI) in IterativeFullatom. When non_local evaluation is activated the method gen_evaluation_output is called for each batch to add the computation of the scores and energies used for decoy selection. These functions are called with an instance of Batch as the only parameter, the generating functions add specific flags to the flag-file provided by Batch and add special input-files to the directory provided by Batch.

In stage V half of of the generated batches start an all-atom refinement from the archived structures. The other 50% of stage V-generated decoys are started from randomly selected decoys from all previous batches. This hedging yields a broader sampling of the all-atom energy function and thus ensures that a converged full-atom ensemble is only found if its all-atom energies are indeed much lower than those of surrounding conformational space.

2.2. NMR Restraints for Benchmark

Experimental chemical shift data was used in all calculations. Experimental or simulated RDC and NOE data was used as indicated in Table 1 of the main text. Details on restraint generation are given below. Experimental NOE data were used for proteins ER541, BtR324B, ARF1, and ALG13, and for 1i1b, 1i1b[2] and 2z2i NOE restraints were simulated.

2.2.1. Simulated RDC restraints

Simulated RDCs were calculated from an ensemble of 20 structures obtained by relaxing the native structure within the Rosetta all-atom energy and adding Gaussian noise of 0.3Hz amplitude relative to a typical tensor amplitude of D_a=10Hz . The structural variation in the RDC generating ensemble renders the simulated data more realistic: even before noise is added no single structure can be fitted to the RDCs with $Q < 10\%$; the native structure fits the resulting RDCs with a *Q*-factor of 17-30%. The noise amplitude of 0.3Hz used here might be unrealistically small in the context of the larger structures and noise estimates around +/- 1 or even +/-2 Hz might be more realistic. In practice, however, the magnitude of the added noise is relatively unimportant when used within a quadratic restraint potential. Thus the structural noise is far more detrimental to structure determination.

2.2.2. NOE Restraints

a. Experimental

For 2jyx and 2kd7 distance restraints based on experimental NOEs were taken from the published data set (BtR324B, PDB id: 2kd7; ER541, PDB id: 2jyx and ARF1, PDB id: 2k5u). To determine which of those published NOE cross-peaks would be unambiguously and automatically assignable from a 4D NOESY we filtered for HN-HN peaks that were separated from each other by more than the typical tolerance used in automated NOESY assignment for the (1H direct/1H indirect/15N) dimensions (0.03/0.05/0.4 ppm). We have deliberately chosen a more conservative set of tolerances than the usually employed (0.02/0.05/0.2ppm) to make sure that structure calculation from the generated set of sparse NOEs is not easier than during a blind calculation.

b. Simulated

For 1i1b, 2rn2, 2z2i, 1sua and 1g68 NOE distance constraints were generated from the native structure as follows: for each HN-HN distance < 4 Å we generated a constraint with lower bound of 1.5 Å and upper bound of 6 Å. For protein 1i1b we also generated a set of HN-HN NOEs with a cutoff < 5 Å . The results for this data set are reported as 1i1b2. Normally 5-6 Å is the detection limit in protonated samples. For deuterated samples and long NOE mixing times one can see peaks for amide protons up to 7 Å apart. In analogy to our conservative strategy for ER541 and BtR324B we have deliberately chosen the conservative cutoff of 4Å.

2.2.3. RDC Restraint Input Files

a. 2jyx

```
  3  N   3  H -3.6280
  4  N   4  H -9.3940
  5  N   5  H -7.8340
  8  N   8  H -7.8780
 10  N  10  H -7.7780
 11  N  11  H -11.1400
 12  N  12  H -5.3120
 13  N  13  H -5.3920
 14  N  14  H -5.0560
 16  N  16  H -10.1640
 17  N  17  H -8.2040
 22  N  22  H -4.6820
 23  N  23  H -7.8860
 24  N  24  H -11.9500
 27  N  27  H -10.8740
 29  N  29  H 0.5020
 33  N  33  H -16.6080
 34  N  34  H -4.9420
 35  N  35  H -7.8820
 37  N  37  H -9.8440
 38  N  38  H -3.3660
 39  N  39  H -9.4800
 40  N  40  H -11.4720
 43  N  43  H -7.7460
 45  N  45  H -9.5860
 46  N  46  H 2.6160
 48  N  48  H 12.7020
 49  N  49  H 4.0240
 51  N  51  H -6.0340
 52  N  52  H -8.4460
 53  N  53  H -11.9780
 56  N  56  H -2.9400
 57  N  57  H 0.8380
 58  N  58  H 13.3740
```

59	N	59	H	11.3080
60	N	60	H	-8.8420
61	N	61	H	-11.3720
62	N	62	H	1.1740
63	N	63	H	9.9300
64	N	64	H	10.9360
65	N	65	H	-8.3140
66	N	66	H	17.7640
67	N	67	H	10.3380
68	N	68	H	-8.4620
69	N	69	H	15.2000
70	N	70	H	1.2020
71	N	71	H	-6.1980
73	N	73	H	-7.0520
74	N	74	H	5.3080
75	N	75	H	2.2140
76	N	76	H	3.8880
79	N	79	H	-0.9720
80	N	80	H	-3.9920
81	N	81	H	-7.0320
83	N	83	H	-6.1320
84	N	84	H	-0.0120
85	N	85	H	8.4380
86	N	86	H	1.6280
89	N	89	H	-6.8380
90	N	90	H	-9.3620
91	N	91	H	-9.5760
92	N	92	H	-4.7120
93	N	93	H	8.8300
94	N	94	H	5.6300
96	N	96	H	-8.6280
99	N	99	H	6.0660
102	N	102	H	22.4560
104	N	104	H	-11.8900
105	N	105	H	-4.5080
106	N	106	H	1.9760
107	N	107	H	-6.8800
109	N	109	H	10.9020
110	N	110	H	22.0860
112	N	112	H	1.6920
113	N	113	H	12.3020
114	N	114	H	14.7140
115	N	115	H	-7.2500
116	N	116	H	-5.4580
117	N	117	H	-5.3860
118	N	118	H	2.3080
119	N	119	H	4.4760
120	N	120	H	14.1800
121	N	121	H	-2.3640

b. 2kd7

b.i. medium 1

5	H	5	N	0.878
6	H	6	N	7.097
7	H	7	N	-4.349
10	H	10	N	-7.208
11	H	11	N	-0.766
12	H	12	N	-1.884
13	H	13	N	-1.105
15	H	15	N	-2.404
16	H	16	N	-0.332

18	H	18	N	1.532
19	H	19	N	-0.783
28	H	28	N	-3.398
29	H	29	N	-5.943
30	H	30	N	-3.594
31	H	31	N	-1.860
32	H	32	N	1.065
33	H	33	N	-6.784
34	H	34	N	-2.337
35	H	35	N	6.988
40	H	40	N	5.523
41	H	41	N	2.676
42	H	42	N	-0.181
43	H	43	N	-0.295
44	H	44	N	-1.379
55	H	55	N	-1.307
56	H	56	N	-1.621
57	H	57	N	-1.489
58	H	58	N	0.232
59	H	59	N	-1.802
61	H	61	N	-3.962
62	H	62	N	-2.436
63	H	63	N	4.146
65	H	65	N	0.323
66	H	66	N	-3.927
67	H	67	N	-4.535
68	H	68	N	-4.373
70	H	70	N	1.051
73	H	73	N	0.319
75	H	75	N	-6.735
83	H	83	N	-6.255
84	H	84	N	-4.867
85	H	85	N	-0.729
86	H	86	N	-1.374
87	H	87	N	-0.470
88	H	88	N	-1.009
90	H	90	N	-3.783
91	H	91	N	-6.332
98	H	98	N	-4.827
100	H	100	N	2.175
102	H	102	N	0.243
103	H	103	N	-0.941
112	H	112	N	-1.921
113	H	113	N	0.931
114	H	114	N	0.893
115	H	115	N	4.811
116	H	116	N	-1.866
117	H	117	N	3.723
118	H	118	N	-1.964
119	H	119	N	-3.870
120	H	120	N	-5.209
121	H	121	N	-7.086
124	H	124	N	-1.272
125	H	125	N	0.388
126	H	126	N	-1.170
127	H	127	N	-0.772
128	H	128	N	-2.409
139	H	139	N	-0.285
140	H	140	N	-2.843
141	H	141	N	-0.771
142	H	142	N	1.240
143	H	143	N	0.389

145	H	145	N	-1.198
146	H	146	N	-2.342
147	H	147	N	-5.694
149	H	149	N	-7.735

b.ii. medium 2

5	H	5	N	-10.223
6	H	6	N	-30.601
7	H	7	N	4.551
11	H	11	N	7.152
12	H	12	N	14.185
13	H	13	N	29.252
15	H	15	N	21.761
16	H	16	N	18.583
18	H	18	N	-9.512
19	H	19	N	-3.031
28	H	28	N	7.279
29	H	29	N	3.883
31	H	31	N	14.711
34	H	34	N	17.393
35	H	35	N	-31.210
40	H	40	N	-25.722
41	H	41	N	1.312
43	H	43	N	25.227
44	H	44	N	29.478
56	H	56	N	11.899
57	H	57	N	26.805
58	H	58	N	19.196
59	H	59	N	25.585
61	H	61	N	11.058
63	H	63	N	-17.257
65	H	65	N	-18.423
67	H	67	N	6.558
68	H	68	N	14.366
70	H	70	N	11.877
73	H	73	N	20.381
75	H	75	N	1.005
83	H	83	N	4.747
84	H	84	N	14.192
85	H	85	N	9.297
86	H	86	N	19.704
87	H	87	N	22.642
88	H	88	N	23.625
90	H	90	N	15.911
91	H	91	N	8.034
98	H	98	N	3.694
100	H	100	N	2.590
112	H	112	N	26.333
113	H	113	N	14.486
114	H	114	N	19.005
115	H	115	N	-7.686
116	H	116	N	-5.584
117	H	117	N	-18.492
118	H	118	N	25.492
119	H	119	N	2.422
120	H	120	N	7.450
121	H	121	N	2.111
124	H	124	N	20.760
126	H	126	N	30.000
128	H	128	N	27.561
139	H	139	N	25.713

```
143   H 143   N  24.560
145   H 145   N   4.975
146   H 146   N  -3.623
147   H 147   N   2.305
149   H 149   N   3.441
150   H 150   N -16.199
```

c. 1i1b

```
2 N 2 H 6.39072
3 N 3 H 2.39428
4 N 4 H 4.89529
5 N 5 H 0.58501
6 N 6 H 4.34939
7 N 7 H 1.74593
8 N 8 H -11.77050
9 N 9 H -3.71675
10 N 10 H -6.01706
11 N 11 H 12.21313
12 N 12 H 2.85739
13 N 13 H 5.68243
14 N 14 H 2.68251
15 N 15 H 11.30584
16 N 16 H -1.48396
17 N 17 H -0.34951
18 N 18 H 6.97161
19 N 19 H -0.35630
20 N 20 H -7.01053
22 N 22 H 1.08152
23 N 23 H -1.93114
24 N 24 H 9.40148
25 N 25 H 1.65733
26 N 26 H 3.15404
27 N 27 H -0.02463
28 N 28 H -0.96655
29 N 29 H -4.36165
30 N 30 H -3.61741
31 N 31 H -6.41555
32 N 32 H 9.34902
33 N 33 H -3.49914
34 N 34 H 9.97243
35 N 35 H 12.25953
36 N 36 H 1.90527
37 N 37 H 9.69166
38 N 38 H -2.47537
39 N 39 H -14.13620
40 N 40 H -6.64714
41 N 41 H -3.15197
42 N 42 H 11.09516
43 N 43 H -1.51674
44 N 44 H 0.74053
45 N 45 H -8.10459
46 N 46 H -13.14200
47 N 47 H -0.02362
48 N 48 H -1.43404
49 N 49 H 8.61226
50 N 50 H 0.84854
51 N 51 H -2.98596
52 N 52 H 15.07108
53 N 53 H 5.14693
54 N 54 H 8.23438
56 N 56 H -2.55943
```

57 N 57 H 0.30543
58 N 58 H 7.38422
59 N 59 H -2.45203
60 N 60 H -1.07524
61 N 61 H 3.10223
62 N 62 H -4.23509
63 N 63 H -12.84034
64 N 64 H -9.71730
65 N 65 H 5.14132
66 N 66 H 2.62946
67 N 67 H -0.03378
68 N 68 H 2.26858
69 N 69 H 5.29595
70 N 70 H 10.63517
71 N 71 H 0.56899
72 N 72 H -14.43071
73 N 73 H -10.49247
74 N 74 H -10.43578
75 N 75 H -14.15590
77 N 77 H 2.64810
78 N 78 H 5.16638
79 N 79 H 10.04316
80 N 80 H 6.72179
81 N 81 H -2.87978
82 N 82 H -2.79852
83 N 83 H 12.63914
84 N 84 H 6.60045
86 N 86 H 0.75814
87 N 87 H 0.30724
88 N 88 H -9.77827
90 N 90 H -0.22101
91 N 91 H -3.55034
92 N 92 H -12.73839
93 N 93 H -3.97495
94 N 94 H -7.37001
95 N 95 H 10.85247
96 N 96 H -1.27671
97 N 97 H 10.49631
98 N 98 H 11.40687
99 N 99 H 0.21087
100 N 100 H 2.01100
101 N 101 H -1.78405
102 N 102 H -1.17840
103 N 103 H 8.77698
104 N 104 H -3.90349
105 N 105 H 6.47499
106 N 106 H 12.01405
107 N 107 H -4.47797
108 N 108 H 7.90607
109 N 109 H 0.10697
110 N 110 H 4.31412
111 N 111 H -2.03309
112 N 112 H -1.57365
113 N 113 H -1.38382
114 N 114 H 9.82455
115 N 115 H 10.91882
117 N 117 H 0.86716
118 N 118 H 2.38090
119 N 119 H 0.85272
120 N 120 H 1.10897
121 N 121 H -8.76030
122 N 122 H -12.62315

```
123 N 123 H -10.28321
124 N 124 H 6.80732
125 N 125 H 14.03762
126 N 126 H 4.47677
127 N 127 H -2.57116
128 N 128 H -4.41530
130 N 130 H -11.11323
131 N 131 H -7.58237
132 N 132 H -5.91508
133 N 133 H 2.19602
134 N 134 H 0.23234
135 N 135 H -6.08194
136 N 136 H 3.02418
137 N 137 H -10.85053
138 N 138 H 6.38151
139 N 139 H 0.08289
140 N 140 H 4.60087
141 N 141 H -11.86752
142 N 142 H -6.81095
143 N 143 H 4.28078
144 N 144 H 6.91522
145 N 145 H -0.54331
146 N 146 H -11.63461
147 N 147 H -6.05659
148 N 148 H -13.69508
149 N 149 H 11.97867
150 N 150 H 9.32606
151 N 151 H 1.32188
```

d. 1i1b_2

<same RDC input file as for 1i1b>

e. 2k5u

```
  2   N     2   H        5.88
  7   N     7   H      -13.11
  9   N     9   H       -1.27
 10   N    10   H       -4.31
 11   N    11   H       16.40
 12   N    12   H       -9.09
 13   N    13   H       13.90
 14   N    14   H        3.01
 15   N    15   H        2.64
 20   N    20   H       -4.82
 21   N    21   H        3.43
 22   N    22   H        2.81
 25   N    25   H       -6.01
 30   N    30   H       -4.82
 33   N    33   H       10.16
 34   N    34   H        7.54
 35   N    35   H        6.09
 37   N    37   H       -9.44
 39   N    39   H       -6.77
 42   N    42   H       -3.73
 43   N    43   H        8.45
 50   N    50   H       -6.09
 52   N    52   H      -12.88
 54   N    54   H        7.24
 55   N    55   H        0.06
 56   N    56   H        6.31
 63   N    63   H       -1.08
 64   N    64   H       -3.45
 67   N    67   H        0.61
```

68	N	68	H	6.78
69	N	69	H	2.74
72	N	72	H	-8.91
74	N	74	H	-10.06
75	N	75	H	-11.72
76	N	76	H	2.50
78	N	78	H	18.03
79	N	79	H	8.28
80	N	80	H	-7.27
81	N	81	H	-12.54
82	N	82	H	6.56
84	N	84	H	22.22
85	N	85	H	-1.67
86	N	86	H	-11.73
87	N	87	H	1.09
89	N	89	H	-8.92
90	N	90	H	-9.06
91	N	91	H	3.63
92	N	92	H	-6.29
93	N	93	H	-10.12
94	N	94	H	-6.07
96	N	96	H	-11.31
97	N	97	H	-14.73
98	N	98	H	19.29
99	N	99	H	9.42
100	N	100	H	-6.10
101	N	101	H	8.64
102	N	102	H	13.31
103	N	103	H	-6.50
104	N	104	H	11.17
105	N	105	H	-7.79
108	N	108	H	-8.51
109	N	109	H	2.56
110	N	110	H	0.91
111	N	111	H	16.02
112	N	112	H	-1.21
113	N	113	H	-11.36
114	N	114	H	-2.80
115	N	115	H	4.87
118	N	118	H	23.17
119	N	119	H	-2.42
120	N	120	H	-4.87
121	N	121	H	21.41
122	N	122	H	19.18
123	N	123	H	16.98
124	N	124	H	22.57
126	N	126	H	16.76
127	N	127	H	22.59
128	N	128	H	17.14
129	N	129	H	2.98
130	N	130	H	1.38
131	N	131	H	-11.73
133	N	133	H	14.06
134	N	134	H	9.72
136	N	136	H	-6.73
140	N	140	H	-6.49
141	N	141	H	0.65
142	N	142	H	-6.60
145	N	145	H	13.52
146	N	146	H	-2.31
147	N	147	H	5.68
148	N	148	H	8.26

149	N	149	H	-13.60
151	N	151	H	2.77
152	N	152	H	4.41
153	N	153	H	-4.96
154	N	154	H	-7.28
156	N	156	H	-4.13
157	N	157	H	-8.12
158	N	158	H	-5.30
159	N	159	H	6.61
160	N	160	H	-2.48
161	N	161	H	-8.44
162	N	162	H	8.45
163	N	163	H	-2.11

f. 2z2i

2 N 2 H -13.22150
3 N 3 H -31.27997
5 N 5 H -2.42659
6 N 6 H -23.74319
7 N 7 H -14.67118
8 N 8 H -27.21860
9 N 9 H -17.33336
10 N 10 H -13.35288
11 N 11 H 16.44735
12 N 12 H 18.95858
14 N 14 H 13.37829
15 N 15 H -27.77608
16 N 16 H 3.91589
17 N 17 H 5.73694
18 N 18 H -31.15317
19 N 19 H -9.49951
20 N 20 H 1.79071
21 N 21 H 15.17711
22 N 22 H -7.53304
23 N 23 H 22.64798
24 N 24 H 8.26982
25 N 25 H 14.22181
26 N 26 H 15.45572
27 N 27 H 13.43209
28 N 28 H 10.70775
29 N 29 H 13.03223
30 N 30 H 17.48356
31 N 31 H 10.61260
32 N 32 H 12.66281
33 N 33 H 13.19406
34 N 34 H 14.28022
35 N 35 H 8.96339
36 N 36 H 10.12647
37 N 37 H 17.94709
38 N 38 H -10.78994
39 N 39 H 21.42204
40 N 40 H 15.04667
41 N 41 H -27.15577
42 N 42 H -23.38187
43 N 43 H 18.04223
44 N 44 H 13.98382
45 N 45 H 5.33425
46 N 46 H 4.59410
47 N 47 H 17.01947
48 N 48 H 6.16133
49 N 49 H 16.44381

50 N 50 H -21.67464
51 N 51 H -12.56712
52 N 52 H -23.13396
53 N 53 H -4.03790
54 N 54 H 5.50723
55 N 55 H 16.04771
56 N 56 H 10.33861
57 N 57 H -14.52459
58 N 58 H 22.48349
59 N 59 H 0.83081
60 N 60 H 2.98506
61 N 61 H -20.98924
62 N 62 H 2.71397
63 N 63 H -12.28596
64 N 64 H -10.33312
66 N 66 H 22.30675
67 N 67 H -5.12377
68 N 68 H 1.30698
69 N 69 H 14.13068
70 N 70 H -4.18261
71 N 71 H 10.10933
72 N 72 H 18.79551
73 N 73 H 18.96129
74 N 74 H 19.34731
75 N 75 H 13.54399
76 N 76 H 19.00834
77 N 77 H 10.13606
79 N 79 H 19.55245
80 N 80 H 17.13483
81 N 81 H 7.37598
82 N 82 H 18.18945
83 N 83 H 20.28922
84 N 84 H -10.90945
85 N 85 H -15.45012
86 N 86 H 8.19073
88 N 88 H 20.87398
89 N 89 H 11.86432
90 N 90 H -9.78400
91 N 91 H -7.86747
92 N 92 H -29.12225
93 N 93 H -18.48410
94 N 94 H -25.22773
95 N 95 H -14.93364
96 N 96 H 13.02543
97 N 97 H 17.33407
98 N 98 H 18.53071
99 N 99 H 0.39312
100 N 100 H -27.58619
101 N 101 H 19.90436
102 N 102 H 5.25821
103 N 103 H 12.65751
104 N 104 H 4.74695
105 N 105 H -10.62764
106 N 106 H -19.95868
107 N 107 H -25.69729
108 N 108 H -31.64214
109 N 109 H -28.89925
110 N 110 H 3.84838
111 N 111 H -3.30911
112 N 112 H 11.33800
113 N 113 H -5.83614
114 N 114 H 17.08492

115 N 115 H -21.70252
116 N 116 H 14.51043
117 N 117 H 21.58880
118 N 118 H 21.93164
119 N 119 H 18.51094
120 N 120 H 18.42168
121 N 121 H 21.80864
122 N 122 H 22.21040
123 N 123 H 14.69217
124 N 124 H 21.89792
125 N 125 H 21.36227
126 N 126 H 1.39552
127 N 127 H -16.28392
128 N 128 H -16.20445
129 N 129 H 16.24792
130 N 130 H 17.97131
131 N 131 H 6.24479
132 N 132 H -32.65418
133 N 133 H -25.09342
134 N 134 H -20.30873
135 N 135 H -4.16459
136 N 136 H -2.92450
137 N 137 H 7.65507
138 N 138 H 0.71040
139 N 139 H -0.03590
142 N 142 H 9.39489
143 N 143 H 9.85048
144 N 144 H -27.16977
145 N 145 H -33.81651
147 N 147 H 11.32286
148 N 148 H 0.41864
149 N 149 H -7.08476
150 N 150 H 5.33451
151 N 151 H 4.89822
152 N 152 H -30.49604
153 N 153 H -2.15987
154 N 154 H -1.65903
155 N 155 H -29.05268
157 N 157 H 13.42262
158 N 158 H 4.91202
159 N 159 H 14.20085
160 N 160 H 18.60660
161 N 161 H 9.59206
162 N 162 H -5.32334
164 N 164 H 19.60042
165 N 165 H 5.07268
166 N 166 H 5.20692
167 N 167 H 19.88966
168 N 168 H 12.59231
169 N 169 H -5.46051
170 N 170 H 8.67825
171 N 171 H 16.71114
172 N 172 H 3.18577
173 N 173 H -4.21729
174 N 174 H 12.77306
175 N 175 H 12.84922
176 N 176 H -6.02342
177 N 177 H 7.04555
178 N 178 H 16.82418
179 N 179 H 2.67463

g. 2jzc

7	N	7	H	4.00
8	N	8	H	13.20
9	N	9	H	7.10
10	N	10	H	12.10
11	N	11	H	3.80
12	N	12	H	8.00
20	N	20	H	7.10
21	N	21	H	-7.70
23	N	23	H	-0.20
24	N	24	H	5.40
25	N	25	H	-5.60
26	N	26	H	2.60
27	N	27	H	10.30
28	N	28	H	-2.50
29	N	29	H	-1.30
30	N	30	H	-5.60
31	N	31	H	-0.70
33	N	33	H	-1.20
34	N	34	H	0.40
35	N	35	H	-0.90
36	N	36	H	-1.50
37	N	37	H	-1.20
38	N	38	H	-2.20
39	N	39	H	-10.50
40	N	40	H	-0.50
42	N	42	H	14.50
43	N	43	H	7.40
44	N	44	H	11.50
60	N	60	H	4.60
61	N	61	H	-4.90
62	N	62	H	-2.00
63	N	63	H	6.20
64	N	64	H	5.20
65	N	65	H	4.00
66	N	66	H	11.40
67	N	67	H	-3.00
72	N	72	H	6.50
73	N	73	H	-0.70
74	N	74	H	6.30
81	N	81	H	-7.70
82	N	82	H	10.00
83	N	83	H	4.60
84	N	84	H	12.90
85	N	85	H	5.80
86	N	86	H	2.30
87	N	87	H	0.30
91	N	91	H	5.10
92	N	92	H	4.50
93	N	93	H	12.60
94	N	94	H	6.60
95	N	95	H	12.10
101	N	101	H	2.60
102	N	102	H	-3.10
103	N	103	H	-8.00
104	N	104	H	-3.20
106	N	106	H	-1.80
107	N	107	H	-7.00
108	N	108	H	-5.60
109	N	109	H	1.00
112	N	112	H	-1.50

```
113   N   113   H        9.80
114   N   114   H        6.60
115   N   115   H       -0.80
116   N   116   H        1.10
120   N   120   H       -1.40
121   N   121   H       -3.00
122   N   122   H        1.40
123   N   123   H       -5.30
124   N   124   H       -0.70
125   N   125   H       -3.50
126   N   126   H      -16.10
128   N   128   H       -1.10
129   N   129   H        2.70
133   N   133   H        6.00
134   N   134   H        1.10
135   N   135   H       -7.50
137   N   137   H       -3.80
147   N   147   H       -1.90
148   N   148   H        2.60
149   N   149   H       -3.90
150   N   150   H       -0.20
151   N   151   H       -2.10
152   N   152   H        2.60
153   N   153   H       -1.80
154   N   154   H       -3.30
155   N   155   H       -1.20
156   N   156   H       -4.30
157   N   157   H        4.20
158   N   158   H        5.80
159   N   159   H        2.30
160   N   160   H       -6.00
162   N   162   H       -7.60
164   N   164   H        1.70
165   N   165   H       -1.50
166   N   166   H       -2.10
167   N   167   H       -1.00
168   N   168   H        2.70
169   N   169   H       -4.40
171   N   171   H        1.00
172   N   172   H       -1.50
173   N   173   H       -2.20
174   N   174   H       -0.40
176   N   176   H       -7.80
177   N   177   H       -3.90
178   N   178   H        0.10
179   N   179   H        0.00
180   N   180   H        6.10
190   N   190   H       -2.90
191   N   191   H       -3.00
192   N   192   H       -8.40
193   N   193   H       -7.50
194   N   194   H       -4.40
195   N   195   H       -5.40
196   N   196   H       -9.40
197   N   197   H       -6.50
```

2.2.4. NOE Restraint Input Files

a. 2jyx

```
AtomPair  H 73 H  115 BOUNDED 1.5 6.27 0.59 NOE ;rawdata 5.980000
AtomPair  H 69 H   85 BOUNDED 1.5 4.70 0.44 NOE ;rawdata 4.480000
AtomPair  H 71 H   85 BOUNDED 1.5 5.21 0.49 NOE ;rawdata 4.970000
```

```
AtomPair  H 84 H   92 BOUNDED 1.5 4.41 0.42 NOE ;rawdata 4.200000
AtomPair  H 91 H  105 BOUNDED 1.5 6.30 0.60 NOE ;rawdata 6.000000
AtomPair  H 68 H   85 BOUNDED 1.5 5.52 0.52 NOE ;rawdata 5.260000
AtomPair  H 58 H  119 BOUNDED 1.5 4.04 0.38 NOE ;rawdata 3.850000
AtomPair  H 70 H  118 BOUNDED 1.5 4.48 0.42 NOE ;rawdata 4.270000
AtomPair  H 93 H  101 BOUNDED 1.5 4.50 0.42 NOE ;rawdata 4.290000
AtomPair  H 74 H  115 BOUNDED 1.5 5.00 0.47 NOE ;rawdata 4.770000
AtomPair  H 71 H   83 BOUNDED 1.5 4.49 0.42 NOE ;rawdata 4.280000
AtomPair  H 22 H   29 BOUNDED 1.5 6.01 0.57 NOE ;rawdata 5.730000
AtomPair  H 95 H   99 BOUNDED 1.5 5.34 0.50 NOE ;rawdata 5.090000
AtomPair  H 49 H   52 BOUNDED 1.5 5.84 0.55 NOE ;rawdata 5.570000
AtomPair  H 95 H  101 BOUNDED 1.5 5.28 0.50 NOE ;rawdata 5.030000
AtomPair  H 19 H   28 BOUNDED 1.5 4.92 0.46 NOE ;rawdata 4.690000
```

b. 2kd7

```
AtomPair H  13 H  59 BOUNDED 1.5 5.76 0.55 NOE ;rawdata 5.49
AtomPair H  28 H  41 BOUNDED 1.5 4.49 0.43 NOE ;rawdata 4.28
AtomPair H  19 H  29 BOUNDED 1.5 5.97 0.57 NOE ;rawdata 5.69
AtomPair H  29 H  43 BOUNDED 1.5 5.55 0.53 NOE ;rawdata 5.29
AtomPair H  28 H  42 BOUNDED 1.5 5.65 0.54 NOE ;rawdata 5.38
AtomPair H  44 H 138 BOUNDED 1.5 4.59 0.44 NOE ;rawdata 4.37
AtomPair H  22 H  47 BOUNDED 1.5 5.95 0.57 NOE ;rawdata 5.67
AtomPair H  57 H 128 BOUNDED 1.5 5.96 0.57 NOE ;rawdata 5.68
AtomPair H  13 H  58 BOUNDED 1.5 5.59 0.53 NOE ;rawdata 5.32
AtomPair H  88 H 100 BOUNDED 1.5 5.12 0.49 NOE ;rawdata 4.88
AtomPair H  90 H 100 BOUNDED 1.5 4.89 0.47 NOE ;rawdata 4.66
AtomPair H  90 H  98 BOUNDED 1.5 3.99 0.38 NOE ;rawdata 3.80
AtomPair H  88 H 103 BOUNDED 1.5 6.02 0.57 NOE ;rawdata 5.73
AtomPair H  86 H 103 BOUNDED 1.5 3.98 0.38 NOE ;rawdata 3.79
AtomPair H  67 H 120 BOUNDED 1.5 5.97 0.57 NOE ;rawdata 5.69
AtomPair H  57 H 126 BOUNDED 1.5 4.15 0.40 NOE ;rawdata 3.95
AtomPair H  59 H 126 BOUNDED 1.5 5.21 0.50 NOE ;rawdata 4.96
AtomPair H  44 H 139 BOUNDED 1.5 5.66 0.54 NOE ;rawdata 5.39
AtomPair H   5 H 145 BOUNDED 1.5 5.13 0.49 NOE ;rawdata 4.89
AtomPair H  68 H 146 BOUNDED 1.5 5.27 0.50 NOE ;rawdata 5.02
AtomPair H  68 H 147 BOUNDED 1.5 5.39 0.51 NOE ;rawdata 5.13
```

c. 1i1b

```
AtomPair H   2 H  44 BOUNDED 1.5 6.00 0.40 SIM_NOE ;rawdata 0.00
AtomPair H   4 H  42 BOUNDED 1.5 6.00 0.40 SIM_NOE ;rawdata 0.00
AtomPair H   6 H  40 BOUNDED 1.5 6.00 0.40 SIM_NOE ;rawdata 0.00
AtomPair H   7 H 147 BOUNDED 1.5 6.00 0.40 SIM_NOE ;rawdata 0.00
AtomPair H   9 H 145 BOUNDED 1.5 6.00 0.40 SIM_NOE ;rawdata 0.00
AtomPair H  11 H 143 BOUNDED 1.5 6.00 0.40 SIM_NOE ;rawdata 0.00
AtomPair H  15 H  27 BOUNDED 1.5 6.00 0.40 SIM_NOE ;rawdata 0.00
AtomPair H  15 H  29 BOUNDED 1.5 6.00 0.40 SIM_NOE ;rawdata 0.00
AtomPair H  17 H  25 BOUNDED 1.5 6.00 0.40 SIM_NOE ;rawdata 0.00
AtomPair H  19 H  25 BOUNDED 1.5 6.00 0.40 SIM_NOE ;rawdata 0.00
AtomPair H  24 H 130 BOUNDED 1.5 6.00 0.40 SIM_NOE ;rawdata 0.00
AtomPair H  26 H 128 BOUNDED 1.5 6.00 0.40 SIM_NOE ;rawdata 0.00
AtomPair H  41 H  59 BOUNDED 1.5 6.00 0.40 SIM_NOE ;rawdata 0.00
AtomPair H  41 H  61 BOUNDED 1.5 6.00 0.40 SIM_NOE ;rawdata 0.00
AtomPair H  43 H  57 BOUNDED 1.5 6.00 0.40 SIM_NOE ;rawdata 0.00
AtomPair H  45 H  57 BOUNDED 1.5 6.00 0.40 SIM_NOE ;rawdata 0.00
AtomPair H  46 H  93 BOUNDED 1.5 6.00 0.40 SIM_NOE ;rawdata 0.00
AtomPair H  54 H 101 BOUNDED 1.5 6.00 0.40 SIM_NOE ;rawdata 0.00
AtomPair H  56 H  99 BOUNDED 1.5 6.00 0.40 SIM_NOE ;rawdata 0.00
AtomPair H  56 H 101 BOUNDED 1.5 6.00 0.40 SIM_NOE ;rawdata 0.00
AtomPair H  66 H  81 BOUNDED 1.5 6.00 0.40 SIM_NOE ;rawdata 0.00
AtomPair H  68 H  79 BOUNDED 1.5 6.00 0.40 SIM_NOE ;rawdata 0.00
AtomPair H  70 H  77 BOUNDED 1.5 6.00 0.40 SIM_NOE ;rawdata 0.00
```

```
AtomPair H  78 H 132 BOUNDED 1.5 6.00 0.40 SIM_NOE ;rawdata 0.00
AtomPair H 100 H 111 BOUNDED 1.5 6.00 0.40 SIM_NOE ;rawdata 0.00
AtomPair H 102 H 109 BOUNDED 1.5 6.00 0.40 SIM_NOE ;rawdata 0.00
AtomPair H 108 H 144 BOUNDED 1.5 6.00 0.40 SIM_NOE ;rawdata 0.00
AtomPair H 110 H 120 BOUNDED 1.5 6.00 0.40 SIM_NOE ;rawdata 0.00
AtomPair H 112 H 118 BOUNDED 1.5 6.00 0.40 SIM_NOE ;rawdata 0.00
AtomPair H 119 H 133 BOUNDED 1.5 6.00 0.40 SIM_NOE ;rawdata 0.00
AtomPair H 121 H 131 BOUNDED 1.5 6.00 0.40 SIM_NOE ;rawdata 0.00
AtomPair H 121 H 133 BOUNDED 1.5 6.00 0.40 SIM_NOE ;rawdata 0.00
AtomPair H 122 H 141 BOUNDED 1.5 6.00 0.40 SIM_NOE ;rawdata 0.00
```

d. 1i1b_2

```
AtomPair H   2 H  44 BOUNDED 1.5 6.00 0.40 SIM_NOE ;rawdata 0.00
AtomPair H   3 H  44 BOUNDED 1.5 6.00 0.40 SIM_NOE ;rawdata 0.00
AtomPair H   4 H  42 BOUNDED 1.5 6.00 0.40 SIM_NOE ;rawdata 0.00
AtomPair H   5 H  42 BOUNDED 1.5 6.00 0.40 SIM_NOE ;rawdata 0.00
AtomPair H   5 H 149 BOUNDED 1.5 6.00 0.40 SIM_NOE ;rawdata 0.00
AtomPair H   6 H  40 BOUNDED 1.5 6.00 0.40 SIM_NOE ;rawdata 0.00
AtomPair H   6 H  42 BOUNDED 1.5 6.00 0.40 SIM_NOE ;rawdata 0.00
AtomPair H   6 H 149 BOUNDED 1.5 6.00 0.40 SIM_NOE ;rawdata 0.00
AtomPair H   7 H 147 BOUNDED 1.5 6.00 0.40 SIM_NOE ;rawdata 0.00
AtomPair H   7 H 149 BOUNDED 1.5 6.00 0.40 SIM_NOE ;rawdata 0.00
AtomPair H   8 H  16 BOUNDED 1.5 6.00 0.40 SIM_NOE ;rawdata 0.00
AtomPair H   9 H 145 BOUNDED 1.5 6.00 0.40 SIM_NOE ;rawdata 0.00
AtomPair H  10 H 145 BOUNDED 1.5 6.00 0.40 SIM_NOE ;rawdata 0.00
AtomPair H  11 H 143 BOUNDED 1.5 6.00 0.40 SIM_NOE ;rawdata 0.00
AtomPair H  15 H  27 BOUNDED 1.5 6.00 0.40 SIM_NOE ;rawdata 0.00
AtomPair H  15 H  28 BOUNDED 1.5 6.00 0.40 SIM_NOE ;rawdata 0.00
AtomPair H  15 H  29 BOUNDED 1.5 6.00 0.40 SIM_NOE ;rawdata 0.00
AtomPair H  16 H  38 BOUNDED 1.5 6.00 0.40 SIM_NOE ;rawdata 0.00
AtomPair H  17 H  25 BOUNDED 1.5 6.00 0.40 SIM_NOE ;rawdata 0.00
AtomPair H  17 H  27 BOUNDED 1.5 6.00 0.40 SIM_NOE ;rawdata 0.00
AtomPair H  17 H  38 BOUNDED 1.5 6.00 0.40 SIM_NOE ;rawdata 0.00
AtomPair H  18 H  25 BOUNDED 1.5 6.00 0.40 SIM_NOE ;rawdata 0.00
AtomPair H  18 H  38 BOUNDED 1.5 6.00 0.40 SIM_NOE ;rawdata 0.00
AtomPair H  19 H  25 BOUNDED 1.5 6.00 0.40 SIM_NOE ;rawdata 0.00
AtomPair H  24 H 130 BOUNDED 1.5 6.00 0.40 SIM_NOE ;rawdata 0.00
AtomPair H  25 H 130 BOUNDED 1.5 6.00 0.40 SIM_NOE ;rawdata 0.00
AtomPair H  26 H 127 BOUNDED 1.5 6.00 0.40 SIM_NOE ;rawdata 0.00
AtomPair H  26 H 128 BOUNDED 1.5 6.00 0.40 SIM_NOE ;rawdata 0.00
AtomPair H  26 H 130 BOUNDED 1.5 6.00 0.40 SIM_NOE ;rawdata 0.00
AtomPair H  27 H 127 BOUNDED 1.5 6.00 0.40 SIM_NOE ;rawdata 0.00
AtomPair H  28 H 127 BOUNDED 1.5 6.00 0.40 SIM_NOE ;rawdata 0.00
AtomPair H  39 H  61 BOUNDED 1.5 6.00 0.40 SIM_NOE ;rawdata 0.00
AtomPair H  40 H  61 BOUNDED 1.5 6.00 0.40 SIM_NOE ;rawdata 0.00
AtomPair H  41 H  59 BOUNDED 1.5 6.00 0.40 SIM_NOE ;rawdata 0.00
AtomPair H  41 H  60 BOUNDED 1.5 6.00 0.40 SIM_NOE ;rawdata 0.00
AtomPair H  41 H  61 BOUNDED 1.5 6.00 0.40 SIM_NOE ;rawdata 0.00
AtomPair H  42 H  59 BOUNDED 1.5 6.00 0.40 SIM_NOE ;rawdata 0.00
AtomPair H  43 H  57 BOUNDED 1.5 6.00 0.40 SIM_NOE ;rawdata 0.00
AtomPair H  43 H  59 BOUNDED 1.5 6.00 0.40 SIM_NOE ;rawdata 0.00
AtomPair H  45 H  56 BOUNDED 1.5 6.00 0.40 SIM_NOE ;rawdata 0.00
AtomPair H  45 H  57 BOUNDED 1.5 6.00 0.40 SIM_NOE ;rawdata 0.00
AtomPair H  46 H  92 BOUNDED 1.5 6.00 0.40 SIM_NOE ;rawdata 0.00
AtomPair H  46 H  93 BOUNDED 1.5 6.00 0.40 SIM_NOE ;rawdata 0.00
AtomPair H  54 H 101 BOUNDED 1.5 6.00 0.40 SIM_NOE ;rawdata 0.00
AtomPair H  54 H 102 BOUNDED 1.5 6.00 0.40 SIM_NOE ;rawdata 0.00
AtomPair H  56 H  99 BOUNDED 1.5 6.00 0.40 SIM_NOE ;rawdata 0.00
AtomPair H  56 H 100 BOUNDED 1.5 6.00 0.40 SIM_NOE ;rawdata 0.00
AtomPair H  56 H 101 BOUNDED 1.5 6.00 0.40 SIM_NOE ;rawdata 0.00
AtomPair H  57 H  99 BOUNDED 1.5 6.00 0.40 SIM_NOE ;rawdata 0.00
```

```
AtomPair H  58 H  67 BOUNDED 1.5 6.00 0.40 SIM_NOE ;rawdata 0.00
AtomPair H  66 H  81 BOUNDED 1.5 6.00 0.40 SIM_NOE ;rawdata 0.00
AtomPair H  66 H  82 BOUNDED 1.5 6.00 0.40 SIM_NOE ;rawdata 0.00
AtomPair H  67 H  81 BOUNDED 1.5 6.00 0.40 SIM_NOE ;rawdata 0.00
AtomPair H  68 H  79 BOUNDED 1.5 6.00 0.40 SIM_NOE ;rawdata 0.00
AtomPair H  68 H  81 BOUNDED 1.5 6.00 0.40 SIM_NOE ;rawdata 0.00
AtomPair H  69 H  79 BOUNDED 1.5 6.00 0.40 SIM_NOE ;rawdata 0.00
AtomPair H  69 H  97 BOUNDED 1.5 6.00 0.40 SIM_NOE ;rawdata 0.00
AtomPair H  70 H  77 BOUNDED 1.5 6.00 0.40 SIM_NOE ;rawdata 0.00
AtomPair H  70 H  79 BOUNDED 1.5 6.00 0.40 SIM_NOE ;rawdata 0.00
AtomPair H  71 H  77 BOUNDED 1.5 6.00 0.40 SIM_NOE ;rawdata 0.00
AtomPair H  78 H 132 BOUNDED 1.5 6.00 0.40 SIM_NOE ;rawdata 0.00
AtomPair H  98 H 113 BOUNDED 1.5 6.00 0.40 SIM_NOE ;rawdata 0.00
AtomPair H  99 H 113 BOUNDED 1.5 6.00 0.40 SIM_NOE ;rawdata 0.00
AtomPair H 100 H 111 BOUNDED 1.5 6.00 0.40 SIM_NOE ;rawdata 0.00
AtomPair H 100 H 112 BOUNDED 1.5 6.00 0.40 SIM_NOE ;rawdata 0.00
AtomPair H 100 H 113 BOUNDED 1.5 6.00 0.40 SIM_NOE ;rawdata 0.00
AtomPair H 102 H 109 BOUNDED 1.5 6.00 0.40 SIM_NOE ;rawdata 0.00
AtomPair H 102 H 110 BOUNDED 1.5 6.00 0.40 SIM_NOE ;rawdata 0.00
AtomPair H 102 H 111 BOUNDED 1.5 6.00 0.40 SIM_NOE ;rawdata 0.00
AtomPair H 108 H 144 BOUNDED 1.5 6.00 0.40 SIM_NOE ;rawdata 0.00
AtomPair H 108 H 145 BOUNDED 1.5 6.00 0.40 SIM_NOE ;rawdata 0.00
AtomPair H 109 H 144 BOUNDED 1.5 6.00 0.40 SIM_NOE ;rawdata 0.00
AtomPair H 110 H 120 BOUNDED 1.5 6.00 0.40 SIM_NOE ;rawdata 0.00
AtomPair H 112 H 118 BOUNDED 1.5 6.00 0.40 SIM_NOE ;rawdata 0.00
AtomPair H 112 H 119 BOUNDED 1.5 6.00 0.40 SIM_NOE ;rawdata 0.00
AtomPair H 112 H 120 BOUNDED 1.5 6.00 0.40 SIM_NOE ;rawdata 0.00
AtomPair H 119 H 133 BOUNDED 1.5 6.00 0.40 SIM_NOE ;rawdata 0.00
AtomPair H 121 H 131 BOUNDED 1.5 6.00 0.40 SIM_NOE ;rawdata 0.00
AtomPair H 121 H 132 BOUNDED 1.5 6.00 0.40 SIM_NOE ;rawdata 0.00
AtomPair H 121 H 133 BOUNDED 1.5 6.00 0.40 SIM_NOE ;rawdata 0.00
AtomPair H 122 H 141 BOUNDED 1.5 6.00 0.40 SIM_NOE ;rawdata 0.00
AtomPair H 122 H 142 BOUNDED 1.5 6.00 0.40 SIM_NOE ;rawdata 0.00
AtomPair H 123 H 141 BOUNDED 1.5 6.00 0.40 SIM_NOE ;rawdata 0.00
AtomPair H 124 H 141 BOUNDED 1.5 6.00 0.40 SIM_NOE ;rawdata 0.00
```

e. 2k5u

```
AtomPair H   9 H  53 BOUNDED 1.5 4.50 0.40 NOE
AtomPair H  11 H  53 BOUNDED 1.5 6.50 0.40 NOE
AtomPair H  28 H  40 BOUNDED 1.5 6.50 0.40 NOE
AtomPair H  38 H  30 BOUNDED 1.5 4.50 0.40 NOE
AtomPair H  39 H  50 BOUNDED 1.5 4.50 0.40 NOE
AtomPair H  40 H  28 BOUNDED 1.5 4.50 0.40 NOE
AtomPair H  50 H  39 BOUNDED 1.5 4.50 0.40 NOE
AtomPair H  53 H   9 BOUNDED 1.5 4.50 0.40 NOE
AtomPair H  53 H  11 BOUNDED 1.5 6.50 0.40 NOE
AtomPair H  79 H 111 BOUNDED 1.5 6.50 0.40 NOE
AtomPair H 111 H  79 BOUNDED 1.5 6.50 0.40 NOE
AtomPair H 112 H 145 BOUNDED 1.5 6.50 0.40 NOE
AtomPair H 113 H 145 BOUNDED 1.5 6.50 0.40 NOE
AtomPair H 119 H  81 BOUNDED 1.5 6.50 0.40 NOE
AtomPair H 143 H 112 BOUNDED 1.5 6.50 0.40 NOE
AtomPair H 143 H 113 BOUNDED 1.5 6.50 0.40 NOE
```

f. 2z2i

```
AtomPair H   5 H  61 BOUNDED 1.5 6.00 0.40 SIM_NOE
AtomPair H   6 H  91 BOUNDED 1.5 6.00 0.40 SIM_NOE
AtomPair H   7 H  61 BOUNDED 1.5 6.00 0.40 SIM_NOE
AtomPair H   8 H  91 BOUNDED 1.5 6.00 0.40 SIM_NOE
AtomPair H   8 H  93 BOUNDED 1.5 6.00 0.40 SIM_NOE
```

```
AtomPair H   9 H  63 BOUNDED 1.5 6.00 0.40 SIM_NOE
AtomPair H  10 H  25 BOUNDED 1.5 6.00 0.40 SIM_NOE
AtomPair H  21 H 152 BOUNDED 1.5 6.00 0.40 SIM_NOE
AtomPair H  25 H  95 BOUNDED 1.5 6.00 0.40 SIM_NOE
AtomPair H  41 H  50 BOUNDED 1.5 6.00 0.40 SIM_NOE
AtomPair H  49 H  64 BOUNDED 1.5 6.00 0.40 SIM_NOE
AtomPair H  51 H  62 BOUNDED 1.5 6.00 0.40 SIM_NOE
AtomPair H  53 H  60 BOUNDED 1.5 6.00 0.40 SIM_NOE
AtomPair H  73 H 121 BOUNDED 1.5 6.00 0.40 SIM_NOE
AtomPair H  90 H 131 BOUNDED 1.5 6.00 0.40 SIM_NOE
AtomPair H  92 H 133 BOUNDED 1.5 6.00 0.40 SIM_NOE
AtomPair H  94 H 133 BOUNDED 1.5 6.00 0.40 SIM_NOE
AtomPair H  94 H 135 BOUNDED 1.5 6.00 0.40 SIM_NOE
AtomPair H  96 H 135 BOUNDED 1.5 6.00 0.40 SIM_NOE
AtomPair H  96 H 137 BOUNDED 1.5 6.00 0.40 SIM_NOE
AtomPair H  99 H 137 BOUNDED 1.5 6.00 0.40 SIM_NOE
AtomPair H  99 H 138 BOUNDED 1.5 6.00 0.40 SIM_NOE
AtomPair H 101 H 138 BOUNDED 1.5 6.00 0.40 SIM_NOE
AtomPair H 103 H 136 BOUNDED 1.5 6.00 0.40 SIM_NOE
AtomPair H 105 H 134 BOUNDED 1.5 6.00 0.40 SIM_NOE
AtomPair H 107 H 132 BOUNDED 1.5 6.00 0.40 SIM_NOE
AtomPair H 109 H 130 BOUNDED 1.5 6.00 0.40 SIM_NOE
```

g. 2jzc

```
AtomPair H   7 H  40 BOUNDED 1.5 7.35 0.70 NOE ;rawdata 7.00
AtomPair H   7 H  41 BOUNDED 1.5 6.16 0.59 NOE ;rawdata 5.87
AtomPair H   7 H 110 BOUNDED 1.5 6.39 0.61 NOE ;rawdata 6.09
AtomPair H   7 H 111 BOUNDED 1.5 4.45 0.42 NOE ;rawdata 4.24
AtomPair H   7 H 112 BOUNDED 1.5 4.30 0.41 NOE ;rawdata 4.10
AtomPair H   8 H  40 BOUNDED 1.5 6.49 0.62 NOE ;rawdata 6.18
AtomPair H   8 H  41 BOUNDED 1.5 4.88 0.47 NOE ;rawdata 4.65
AtomPair H   8 H  42 BOUNDED 1.5 5.12 0.49 NOE ;rawdata 4.88
AtomPair H   8 H  43 BOUNDED 1.5 4.83 0.46 NOE ;rawdata 4.60
AtomPair H   8 H 112 BOUNDED 1.5 6.45 0.61 NOE ;rawdata 6.14
AtomPair H   9 H 111 BOUNDED 1.5 6.39 0.61 NOE ;rawdata 6.09
AtomPair H   9 H 112 BOUNDED 1.5 4.87 0.46 NOE ;rawdata 4.64
AtomPair H   9 H 113 BOUNDED 1.5 5.41 0.52 NOE ;rawdata 5.15
AtomPair H  10 H  43 BOUNDED 1.5 4.53 0.43 NOE ;rawdata 4.31
AtomPair H  10 H  45 BOUNDED 1.5 4.68 0.45 NOE ;rawdata 4.46
AtomPair H  10 H 114 BOUNDED 1.5 5.75 0.55 NOE ;rawdata 5.48
AtomPair H  11 H  45 BOUNDED 1.5 5.89 0.56 NOE ;rawdata 5.61
AtomPair H  11 H 114 BOUNDED 1.5 4.87 0.46 NOE ;rawdata 4.64
AtomPair H  11 H 116 BOUNDED 1.5 5.40 0.51 NOE ;rawdata 5.14
AtomPair H  12 H  45 BOUNDED 1.5 4.86 0.46 NOE ;rawdata 4.63
AtomPair H  40 H  92 BOUNDED 1.5 6.77 0.65 NOE ;rawdata 6.45
AtomPair H  41 H  92 BOUNDED 1.5 6.23 0.59 NOE ;rawdata 5.93
AtomPair H  42 H  92 BOUNDED 1.5 4.68 0.45 NOE ;rawdata 4.46
AtomPair H  44 H  94 BOUNDED 1.5 4.42 0.42 NOE ;rawdata 4.21
AtomPair H  44 H  95 BOUNDED 1.5 6.18 0.59 NOE ;rawdata 5.89
AtomPair H  64 H  85 BOUNDED 1.5 3.84 0.37 NOE ;rawdata 3.66
AtomPair H  64 H  86 BOUNDED 1.5 5.79 0.55 NOE ;rawdata 5.51
AtomPair H  65 H  85 BOUNDED 1.5 7.23 0.69 NOE ;rawdata 6.89
AtomPair H  66 H  83 BOUNDED 1.5 4.51 0.43 NOE ;rawdata 4.30
AtomPair H  66 H  84 BOUNDED 1.5 5.32 0.51 NOE ;rawdata 5.07
AtomPair H  66 H  85 BOUNDED 1.5 6.10 0.58 NOE ;rawdata 5.81
AtomPair H  66 H  95 BOUNDED 1.5 7.35 0.70 NOE ;rawdata 7.00
AtomPair H  81 H  95 BOUNDED 1.5 6.86 0.65 NOE ;rawdata 6.53
AtomPair H  82 H  95 BOUNDED 1.5 4.30 0.41 NOE ;rawdata 4.10
AtomPair H  83 H  94 BOUNDED 1.5 7.35 0.70 NOE ;rawdata 7.00
AtomPair H  83 H  95 BOUNDED 1.5 5.53 0.53 NOE ;rawdata 5.27
AtomPair H  84 H  93 BOUNDED 1.5 3.86 0.37 NOE ;rawdata 3.68
```

```
AtomPair H  84 H  94 BOUNDED 1.5 6.21 0.59 NOE ;rawdata 5.91
AtomPair H  84 H  95 BOUNDED 1.5 6.55 0.62 NOE ;rawdata 6.24
AtomPair H  86 H  92 BOUNDED 1.5 6.37 0.61 NOE ;rawdata 6.07
AtomPair H 113 H 125 BOUNDED 1.5 7.35 0.70 NOE ;rawdata 7.00
AtomPair H 113 H 133 BOUNDED 1.5 4.79 0.46 NOE ;rawdata 4.56
AtomPair H 115 H 122 BOUNDED 1.5 7.35 0.70 NOE ;rawdata 7.00
AtomPair H 115 H 134 BOUNDED 1.5 5.85 0.56 NOE ;rawdata 5.57
AtomPair H 116 H 135 BOUNDED 1.5 5.40 0.51 NOE ;rawdata 5.14
AtomPair H 118 H 135 BOUNDED 1.5 7.35 0.70 NOE ;rawdata 7.00
AtomPair H 133 H 175 BOUNDED 1.5 7.35 0.70 NOE ;rawdata 7.00
AtomPair H 134 H 159 BOUNDED 1.5 4.70 0.45 NOE ;rawdata 4.48
AtomPair H 134 H 160 BOUNDED 1.5 5.20 0.49 NOE ;rawdata 4.95
AtomPair H 134 H 161 BOUNDED 1.5 4.91 0.47 NOE ;rawdata 4.68
AtomPair H 135 H 161 BOUNDED 1.5 4.65 0.44 NOE ;rawdata 4.43
AtomPair H 136 H 161 BOUNDED 1.5 4.07 0.39 NOE ;rawdata 3.88
```
